# A Population-Structured HIV Epidemic in Israel: Roles of Risk and Ethnicity

**DOI:** 10.1371/journal.pone.0135061

**Published:** 2015-08-24

**Authors:** Zehava Grossman, Boaz Avidor, Zohar Mor, Michal Chowers, Itzchak Levy, Eduardo Shahar, Klaris Riesenberg, Zev Sthoeger, Shlomo Maayan, Wei Shao, Margalit Lorber, Karen Olstein-Pops, Daniel Elbirt, Hila Elinav, Ilan Asher, Diana Averbuch, Valery Istomin, Bat Sheva Gottesman, Eynat Kedem, Shirley Girshengorn, Zipi Kra-Oz, Yonat Shemer Avni, Sara Radian Sade, Dan Turner, Frank Maldarelli

**Affiliations:** 1 School of Public Health, Tel-Aviv University, Tel-Aviv, Israel; 2 National Cancer Institute, Frederick, MD, United States of America; 3 Crusaid Kobler AIDS Center, Tel Aviv Sourasky Medical Center, Tel Aviv, Israel; 4 Laboratory of Viruses and Molecular Biology, Tel-Aviv Sourasky Medical Center, Tel Aviv, Israel; 5 Ramla Department of Health, Ministry of Health, Ramla, Israel; 6 Meir Medical Center, Kfar Saba, Israel; 7 Infectious Diseases Unit, Sheba Medical Center, Ramat-Gan, Israel; 8 Rambam Medical Center, Haifa, Israel; 9 Soroka Medical Center, Beer-Sheva, Israel; 10 Kaplan Medical Center, Rehovot, Israel; 11 Hadassah Medical Center, Jerusalem, Israel; 12 Advanced Biomedical Computing Center, SAIC-Frederick, Inc., Frederick National Laboratory for Cancer Research, Frederick, MD, 21702, United States of America; 13 Hillel Yaffe Medical Center, Hadera, Israel; Centro Nacional de Microbiología—Instituto de Salud Carlos III, SPAIN

## Abstract

**Background:**

HIV in Israel started with a subtype-B epidemic among men who have sex with men, followed in the 1980s and 1990s by introductions of subtype C from Ethiopia (predominantly acquired by heterosexual transmission) and subtype A from the former Soviet Union (FSU, most often acquired by intravenous drug use). The epidemic matured over the last 15 years without additional large influx of exogenous infections. Between 2005 and 2013 the number of infected men who have sex with men (MSM) increased 2.9-fold, compared to 1.6-fold and 1.3-fold for intravenous drug users (IVDU) and Ethiopian-origin residents. Understanding contemporary spread is essential for effective public health planning.

**Methods:**

We analyzed demographic and virologic data from 1,427 HIV-infected individuals diagnosed with HIV-I during 1998–2012. HIV phylogenies were reconstructed with maximum-likelihood and Bayesian methods.

**Results:**

Subtype-B viruses, but not A or C, demonstrated a striking number of large clusters with common ancestors having posterior probability ≥0.95, including some suggesting presence of transmission networks. Transmitted drug resistance was highest in subtype B (13%). MSM represented a frequent risk factor in cross-ethnic transmission, demonstrated by the presence of Israeli-born with non-B virus infections and FSU immigrants with non-A subtypes.

**Conclusions:**

Reconstructed phylogenetic trees demonstrated substantial grouping in subtype B, but not in non-MSM subtype-A or in subtype-C, reflecting differences in transmission dynamics linked to HIV transmission categories. Cross-ethnic spread occurred through multiple independent introductions, with MSM playing a prevalent role in the transmission of the virus. Such data provide a baseline to track epidemic trends and will be useful in informing and quantifying efforts to reduce HIV transmission.

## Introduction

HIV initially propagated worldwide in waves of infection characterized by strong founder effects and specific risk factors [1]. In developed areas with ethnically and culturally diverse populations, such as North America [2], Western Europe [3–5], and Israel [6,7], the epidemic spreads with different dynamics across diverse populations, reflecting differences in HIV transmission categories. Characterizing such complex HIV transmission patterns is of intense interest, as particular groups may serve to fuel ongoing epidemic spread more than others, while unanticipated spread across traditional risk and ethnic groups may complicate attempts to track epidemic trends. The HIV epidemic in Israel represents an ideal model to investigate HIV transmission within and across traditional risk and ethnic boundaries.

During the first 20 years of the epidemic, Israel underwent several major introductions of HIV with distinct ethnic- and risk-associated transmission history. Initial appearance of subtype B in men who have sex with men (MSM) as early as 1980 [8] was followed by introduction of subtype C in heterosexuals as part of African (largely Ethiopian) immigration (1991–2000) [9], and subsequently, of subtype A in immigrants from FSU during 1995–2000 with individuals frequently reporting a history of intravenous drug use (IVDU) [9]. The epidemic matured in Israel over the last 15 years without additional large influx of exogenous infections [9–11] but patterns of contemporary HIV spread within Israel among ethnic groups and across HIV transmission risk categories have not been well characterized. We conducted a retrospective analysis of the demographic and virologic characteristics of a large group of antiretroviral-naive individuals newly diagnosed with HIV during 1998–2012.

## Materials and Methods

### Patients

Patients were seen in clinics at seven national AIDS centers in Israel. At genotyping, they were given unique identifiers to preclude analysis of duplicates. Sequences (N = 1,427) were derived from three sources: 1) from 2002 through 2006, individuals with newly diagnosed HIV infection were genotyped as part of the international SPREAD research protocol [3–5] (N = 416); 2) since 2007, all newly diagnosed individuals were genotyped as part of their clinical evaluation (N = 924); 3) 87 samples of individuals with well-documented duration of HIV infection were diagnosed prior to 2002. Samples included 20 children infected via mother to child transmission. Demographic, clinical and virological data were abstracted from the medical record and provided by the treating physicians (Table 1, S1 Fig). In considering the demographics of HIV infection in Israel, we refer to three distinct groups: Israeli nationals of Ethiopian origin, immigrants from FSU, and “Israeli-born”. HIV infection was considered “recent” (<1yr) if seroconversion and/or retroviral syndrome was documented within one year of sample acquisition.

**Table 1:** Demographic characteristics, clinical data and subtypes.

		Subtype A	Subtype B	Subtype C	Total
	N	232	770	425	1427
**Gender** ^**1**^	**Male (%)**	**147 (63.4%**	**727 (94.7%)**	**184 (43.3%)**	**1058 (74%)**
	**Female (%)**	**85 (36.6%)**	**41 (5.3%)**	**241 (56.7%)**	**367 (2%)**
**Age (years)**	**Median**	**33.6**	**34.4**	**36.4**	**34.6**
	**St. Deviation**	**9.8**	**9.8**	**15.3**	**11.8**
	**Range**	**[3–63]**	**[0–78]**	**[0–85]**	**[0–85]**
**Risk**	**Hetero (%)**	**51 (22%)**	**48 (6.2%)**	**376 (88.4%)**	**475 (33%)**
	**IVDU (%)**	**123 (53%)**	**36 (4.7%)**	**10 (2.4%)**	**169 (12%)**
	**MSM (%)**	**29 (12.5%)**	**638 (82.9%)**	**10 (2.4%)**	**677 (48%)**
	**Other (%)**	**29 (12.5%)**	**48 (6.2%)**	**29 (6.8%)**	**106 (7%)**
**Country of Birth**	**Ethiopia (%)**	**3 (1.3%)**	**2 (0.3%)**	**377 (88.9%)**	**383 (27%)**
	**FSU (%)**	**165 (71.1%)**	**100 (13%)**	**10 (2.4%)**	**275 (19%)**
	**Israel (%)**	**41 (17.6%)**	**564 (73.2%)**	**24 (5.4%)**	**628 (44%)**
	**Other /unknown (%)**	**23 (9.9%)**	**104 (13.5%)**	**14 (3.3%)**	**141 (10%)**
**CD4 (cells/μl)**	**Median**	**355**	**386**	**248**	**336**
	**St. Deviation**	**241**	**264**	**305**	**280**
	**25% quartile**	**160**	**266**	**126**	**196**
	**75% quartile**	**500**	**546**	**382**	**498**
	**Range**	**[4–1950]**	**[4–1558]**	**[5–3429]**	**[4–3429]**
**HIV RNA log**_**10**_ **(copies/ml)**	**Median**	**4.48**	**4.67**	**4.7**	**4.66**
	**Std. Deviation**	**5.73**	**6.51**	**6.2**	**6.41**
	**Range**	**[2.00–6.79]**	**[2.60–7.90]**	**[2.45–7.29]**	**[2.00–7.90]**
**Seroconverters (%)**	** **	**4 (1.7%)**	**37 (4.8%)**	**19 (4.5%)**	**60 (4.2%)**

Subtype-B patients had significantly higher CD4 counts than the other groups (*p*<0.0001) suggesting early presentation.

^1^for two B-subtype patients gender was not reported

### Ethics Statement

The retrospective analysis of clinical and laboratory data, which were obtained from the medical charts of HIV-1 patients attending the Hadassah, Sheba and Sourasky Medical Centers, was approved by the respective institutional review boards (IRB) / ethics (Helsinki) committees. Specifically, permission was granted to analyze such data without the need of a signed informed consent by the patients (Sourasky Ethical Committee), or by next of kin, caretakers, or guardians on behalf of the minors/children enrolled in the study (Hadassah Ethical Committee). The samples obtained at the Sheba Medical Center that were used in this study were from patients who had provided written informed consent agreeing to participate in a range of studies.

### Genotyping

Plasma samples were genotyped at the National HIV Reference Laboratory and the Laboratory of Viruses and Molecular Biology, Sourasky Medical Center, with the HIV-I TRUGENE kit (Siemens) [12], using the earliest sample from each patient. Each sequence was manually edited by one of three experts [BA, SG, ZG]. Drug-resistance mutations were identified using the Stanford University HIV Drug-Resistance Database [13,14]. The REGA Subtyping Tool version 3.0 was used for subtyping (www.hivdb.stanford.edu/hiv/) [15]. Recombinants between established subtypes were identified using the REGA Subtyping Tool [15] and SimPlot [16]. Transmitted drug-resistance (TDR) mutations in drug-naive patients were identified according to Bennett *et al*. [17].

### Phylogenetic and Bayesian Molecular-Evolution Analyses

We aligned sequences using ClustalX (MegAlign, Lasergene version 5.01, DNASTAR Inc., Madison WI, USA). Additional reference sequences [18] were included in each dataset analysis. The earliest subtype A, B, and C sequences from different areas with available isolation data were selected from the Los Alamos database as reference sequences to facilitate estimates of the time of the most recent common ancestors (TMRCA). Forty-three transmitted drug-resistance codons [17] were removed from alignment to prevent them from contributing phylogenetic signal, leaving sequences of 791 nucleotides in length. Using jModelTest version 2.1.4 [19], we selected the General Time Reversible model [20], with proportion of invariable sites- and gamma plus invariant sites-distributed rate heterogeneity (GTR+G+I model), as the nucleotide substitution model most appropriate for analysis of our dataset. We constructed maximum likelihood phylogenetic trees [21, 22] of protease and reverse transcriptase sequences with branch support assessed from 1000 bootstrap replicates using MEGA6 version 6.06 [23]. Phylogenetic trees were visualized with FigTree v1.4.0 [24]. Bootstrap ≥80 was considered significant. Alignments were then subjected to Monte-Carlo Markov Chain (MCMC) analyses using Bayesian Evolutionary Analysis Sampling Trees (BEAST) [25] to construct phylogenies and investigate ancestral relationships. The General Time Reversible model of nucleotide substitution with proportion of invariable sites and gamma plus invariant sites-distributed rate heterogeneity and lognormal uncorrelated relaxed clock was used. The MCMC length (burn in 10%) was of 400–600 million states to achieve posterior effective sample size >200 as described [26]. Reconstructed trees were annotated and analyzed with corresponding demographic and epidemiologic data. Clusters were defined as groups of sequences with a posterior probability >0.95 of a recent common ancestor [26]. We use the terms “clusters” or “groups” interchangeably.

### Statistical Analysis

The chi-square test and Fisher’s two-tailed exact test were used for analysis of discrete categorical parameters (*e*.*g*., gender or country of birth) using SPSS (version 21.0). Results are considered statistically significant if *p*<0.05 after Boneferroni correction for multiple comparisons as indicated.

### GenBank Accession Numbers

Sequences were submitted to GenBank previously [5–7,11,27–30] or with this submission (see S1 Text for accession numbers).

## Results

### Spread of HIV Infection in Israel within and across HIV Transmission Categories

As previously described, three distinct large introductions of HIV into Israel have been documented: subtype B from developed countries, from individuals who acquired HIV primarily through MSM exposure, subtype-C from Ethiopia, from individuals who were infected mainly through heterosexual exposure, and subtype-A from individuals from the former Soviet Union who acquired HIV predominantly through intravenous drug use. To characterize patterns of HIV spread in Israel since these introductions, we analyzed viruses from drug-naïve individuals who were newly diagnosed with HIV-1 infection in Israel during the period 1998–2013, after the bulk of influx of A and C infections had occurred. A total of 1,427 individuals with subtypes A (N = 232; 16%), B (N = 770; 54%) and C (N = 425; 30%) were analyzed (Table 1; see S1 Fig for graphical representation).

Analysis of the distribution of HIV subtypes in the immigrant and non-immigrant populations confirmed the expected broad associations with transmission category and country of origin [6,7,9,31]. Individuals with subtype A were mainly (71%) FSU immigrants and were 63% males and principally (53%) IVDU. Subtype-B infected individuals were 95% males, mainly Israeli-born (73%) reporting MSM exposure (83%), whereas subtype-C infected individuals were mainly of Ethiopian-origin (89%) reporting heterosexual HIV exposure (88%). The groups were fairly comparable in age and median HIV viral RNA level; subtype-B patients had significantly higher CD4 counts than the other groups (*p*<0.0001), suggesting earlier presentation to care. A small number of seroconverters were present in each group.

Recently we reported an increasing proportion of newly diagnosed cases among MSM during the period 2003–2010 [6]; between 2005 and 2013 the number of infected MSM living in Israel increased 2.9-fold, compared with increases of only 1.6-fold and 1.3-fold among IVDU and Ethiopian-origin residents, respectively [11] (see S2 Fig, upper panel). Consistent with these observations, we found that the proportion of newly diagnosed individuals reporting MSM exposure in the total population of HIV-infected MSM remained higher than the corresponding proportions in other HIV transmission categories during 2010–2012 (S2 Fig, lower panel).

In addition to the expected distribution of subtypes among ethnic and HIV transmission categories, we also identified a substantial number of infections that did not fit expected ethnic and HIV transmission categorization. Non subtype-B infections in Israeli-born and non subtype-A infections in FSU immigrants were more common than non-C infections in individuals of Ethiopian origin. As shown in Table 1, 10.2% of HIV-infected Israeli-born (N = 65) had non-B subtypes, 6.5% subtype-A (N = 41) and 3.5% subtype-C (N = 24). Of all FSU immigrants identified with HIV, 100 (43%) were infected with subtype B and 10 (3.6%) with C. In contrast, little cross ethnic spread was present in individuals of Ethiopian-origin, as only 5 individuals (1.3%) of Ethiopian-origin were infected with non-C viruses.

Among Israeli-born with subtype-C infection, 11/24 (46%) reported heterosexual exposure, and 6/24 (25%) reported MSM, suggesting multiple risk sources for transmission. Among Israeli-born with subtype-A infection (N = 41), 49% reported MSM exposure risk (20/41) with only 12% reporting IVDU exposure (5/41) and 29% heterosexual HIV exposure risk (12/41). Surprisingly, among subtype-A Israeli-born, MSM exposure exceeded IVDU transmission, which is the most common risk factor reported among FSU immigrants with subtype-A; these data suggest a substantial role for MSM transmission in introduction of subtype A into the Israeli-born population.

Inter-ethnic spread was also evident in FSU immigrants infected with subtype-C (N = 10); these individuals reported MSM contact (N = 2), heterosexual contact (N = 5), and IVDU (N = 1) as risk factors for HIV acquisition, suggesting multiple sources of transmission of subtype C into FSU immigrants.

**Fig 1:**
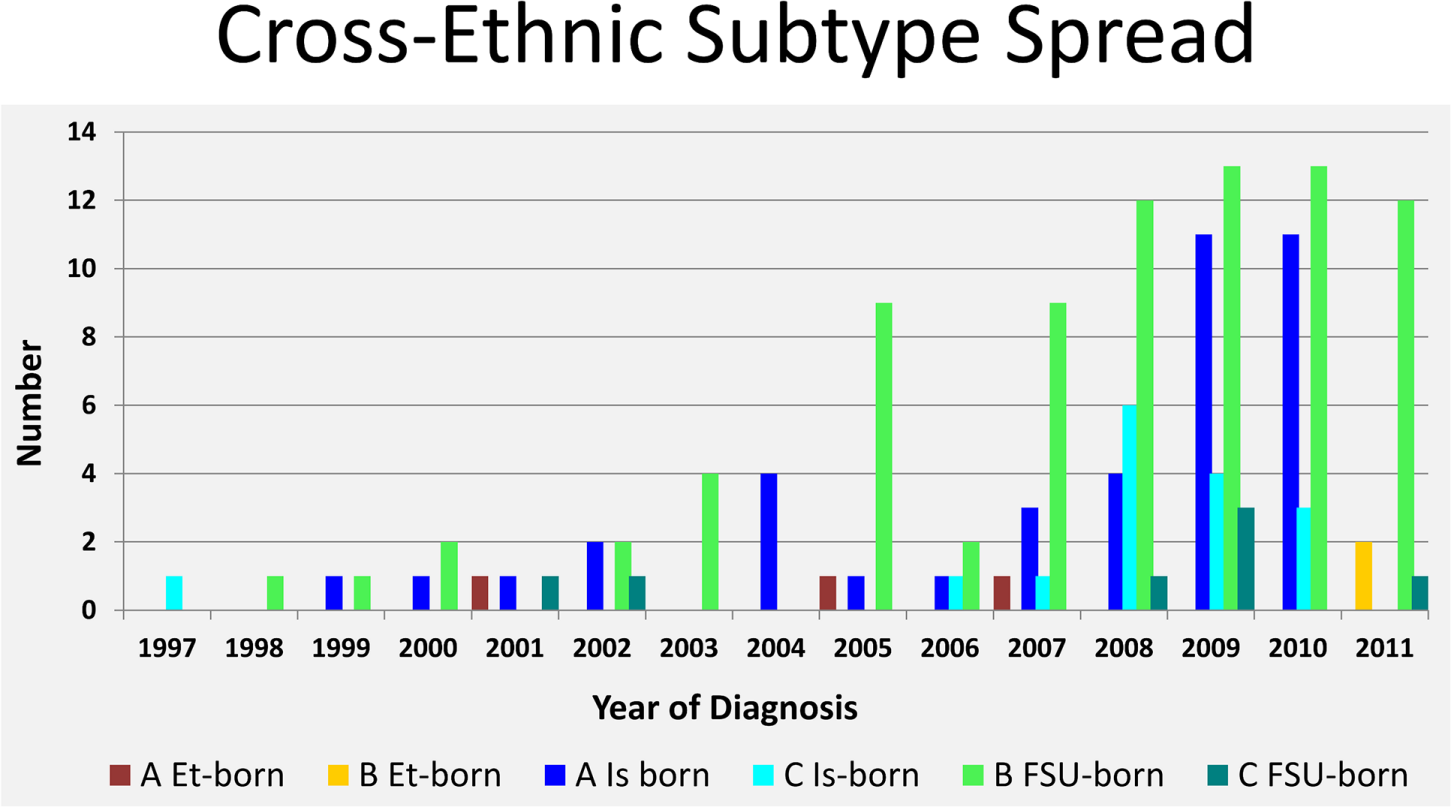
Cross-ethnic subtype spread. Number of individuals diagnosed each year demonstrating cross ethnic spread of HIV, including subtype A or B in Ethiopian born, subtype A or C in Israeli-born, and subtype B or C in FSU immigrants. Et, Ethiopia; FSU, Former Soviet Union; Is, Israel.

We quantified cross-ethnic transmissions over time. As shown in Fig 1, subtype B or C cross-ethnic transmissions were evident as early as 1997; numbers of Israeli-born infected with subtype A or C and FSU immigrants with subtype B or C increased during 2007–2010. The numbers of patients who underwent genotyping were, however, higher at later time points (S2 Fig), so it is possible that our identification of such cross-ethnic transmissions was more sensitive after 2007. In contrast, subtype-B transmission into Ethiopian-origin individuals has been detectable only recently; despite testing 100 or more Ethiopian-origin individuals yearly, subtype B was detected only in two individuals diagnosed after 2010.

### Fine-Structure Analysis of HIV-Infected Populations

Phylogenetic analysis previously indicated emergence of transmission networks involving MSM exposure, particularly since 2007, accounting in part for an increasing rate of new infections over time [6]. The presence of substantial numbers of Israeli-born with non-B virus infections, and FSU immigrants with non-A viruses, demonstrated cross-ethnic transmission; MSM is a frequent HIV risk factor reported for these transmissions. We employed maximum likelihood and Bayesian phylogenetic analyses to further characterize trends of the epidemic in Israel and to specifically investigate whether cross-ethnic transmissions were the product of single or multiple events. Maximum-likelihood analyses of all sequences (N = 1,427, with additional reference sequences) that were grouped according to reported risk factor, also demonstrated cross-ethnic transmission (S3 Fig).

**Fig 2:**
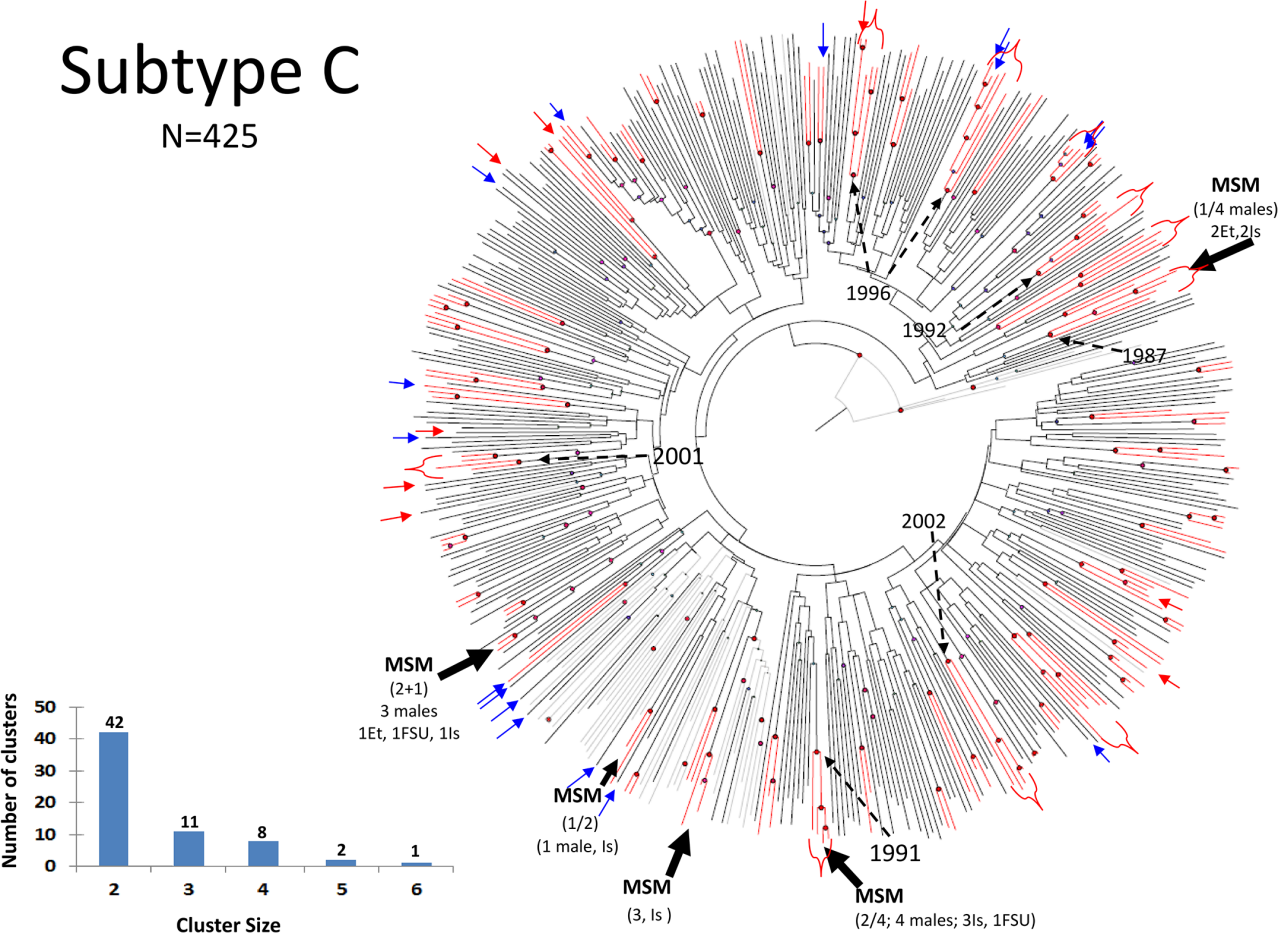
Bayesian evolutionary analysis sampling trees (BEAST) of subtype C. Sequences from patients infected with subtype C viruses were subjected to Monte-Carlo Markov Chain (MCMC) analyses using BEAST to construct phylogenies and investigate ancestral relationships. 47 reference subtype C sequences (http://www.hiv.lanl.gov/) were included. The MCMC length (burn in 10%) was of 400–600 million states to achieve posterior effective sample size (ESS) >200 as described [26]. Red lines represent branches with posterior probability of recent common ancestor ≥0.95. Red-circled nodes represent posterior probability ≥0.95. The largest clusters are marked. Insets describe the number and size of clusters. Israeli-born and FSU-born infected with subtype C are marked with thin blue and red arrows, respectively. Dashed arrows indicate calculated year of selected nodes. FSU, Former Soviet Union; Hetero, heterosexuals; IVDU, intravenous drug users; MSM, men who have sex with men.

Bayesian analysis of 425 subtype-C infections revealed viral clustering (Fig 2), but most of the groups with posterior probability ≥ 0.95 consisted of only two individuals (Fig 2, inset); such small groupings have little epidemiologic utility in tracing. Individuals emigrated from Ethiopia, where HIV epidemic had been longstanding, and data reported here are consistent with the transmission in this group being overwhelmingly heterosexual. In contrast, Israeli-born individuals or FSU-born individuals with subtype C were present either as independent sequences (Fig 2, blue and red arrows, respectively), or mixed in four distinct clusters, each with a different ancestor (Fig 2, Black arrows). The dates of sampling of these clusters are distinct, indicating that clusters were not diagnosed simultaneously and were not the result of specific single-contact transmission (Fig 2). Of interest, all of the four clusters included individuals reporting MSM exposure, and two of those had viruses in common with Ethiopian C infections diagnosed in Israel (Fig 2). The remaining C viruses in Israeli-born had no detectable recent common ancestors with posterior probability ≥ 0.95 in the subtype-C dataset, including reference strains, and were derived from individuals reporting MSM, heterosexual, and IVDU risk factors. These data indicate that introduction of subtype C into Israeli-born is the product of multiple independent events with MSM and heterosexual exposure but involves transmission among MSM.

**Fig 3:**
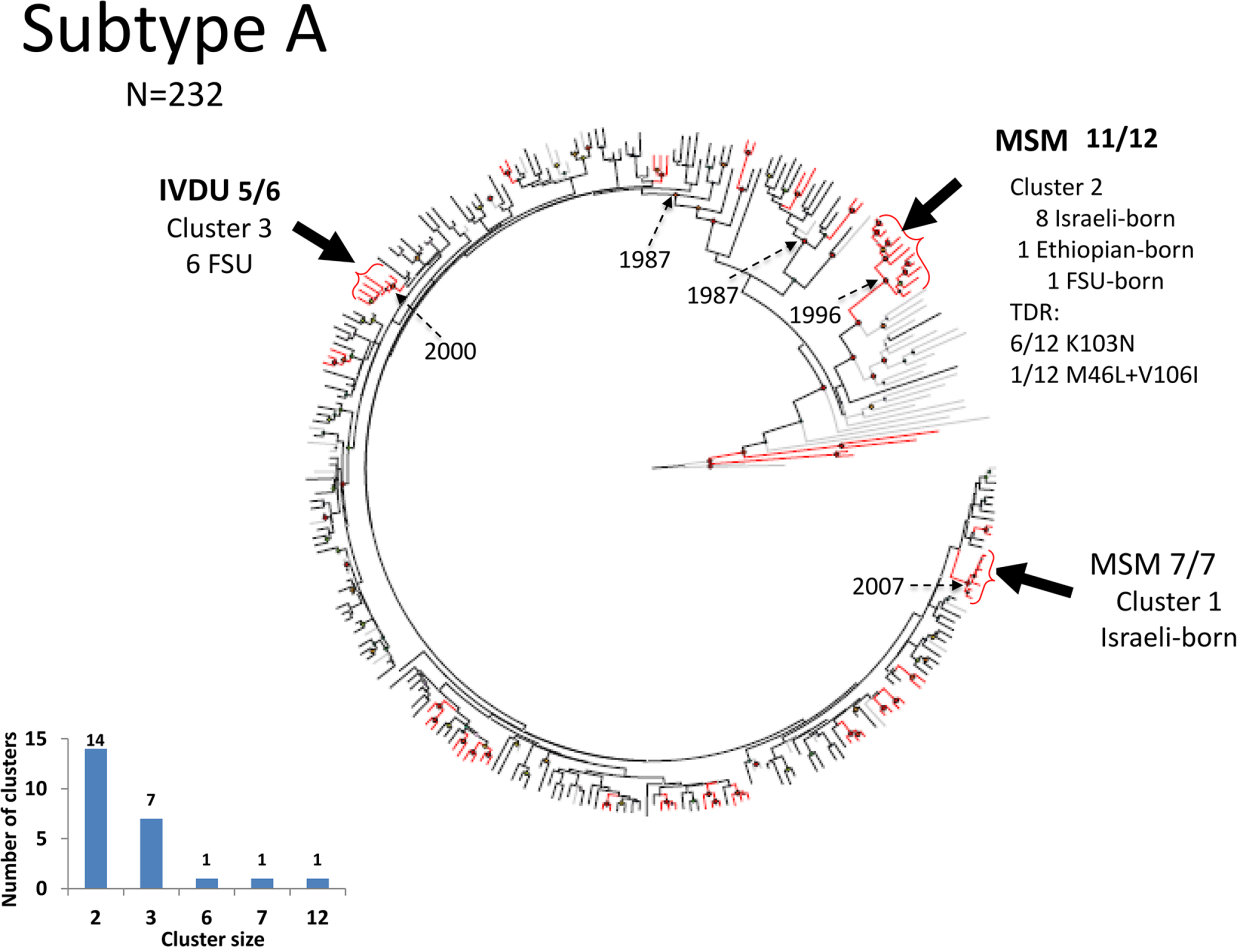
BEAST of subtype A. Analysis of sequences from patients infected with subtype A (Including 61 reference sequences). Notations as in Fig 2.

The subtype-A population included ten clusters with more than two members each, three of which had 6, 7, and 12 members each (Fig 3, brackets). In contrast to the majority of subtype-A infections reporting IVDU exposure for HIV acquisition, the two largest clusters consisted of Israeli-born with either seven MSM (Fig 3, cluster 1) or 11 MSM and one IVDU (Fig 3, cluster 2). Only one FSU immigrant was present in these larger clusters. As the sampling number (N = 232) was substantial relative to the total number of IVDU with HIV, the relatively few clusters of infections suggest that cross-ethnic transmission had numerous sources. In this analysis, 61 reference strains were included, three of which (one each from Ukraine, Georgia, and Belarus) clustered with subtype-A sequences circulating among FSU immigrants in Israel. Subtype-A viruses in Ethiopian-origin Israelis were infrequent (N = 3) and did not have recent common ancestors with posterior probability ≥ 0.95 in our sample.

**Fig 4:**
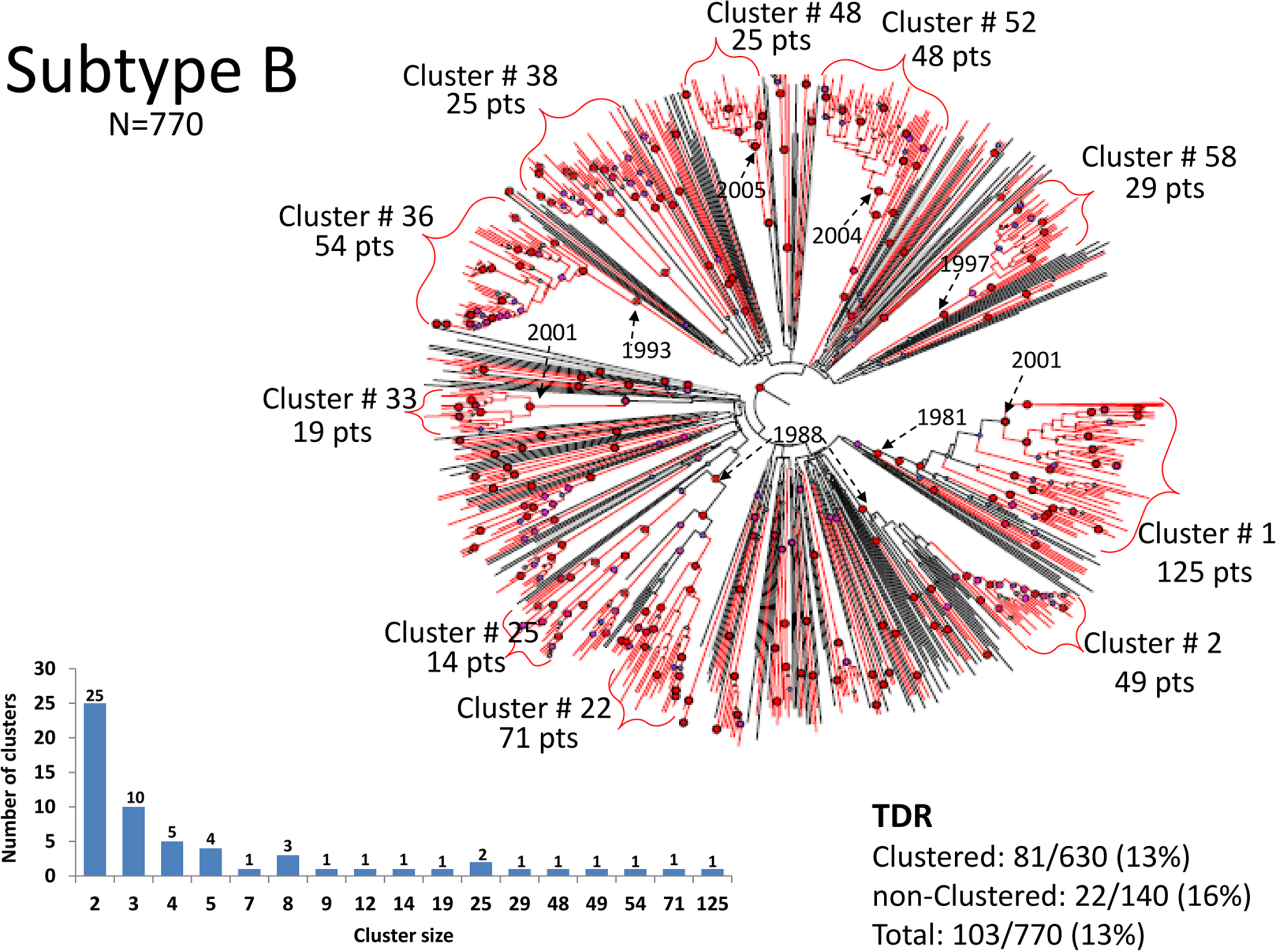
BEAST of subtype B. Analysis of sequences from patients infected with subtype B (Including 30 reference sequences). Notations as in Fig 2.

**Fig 5:**
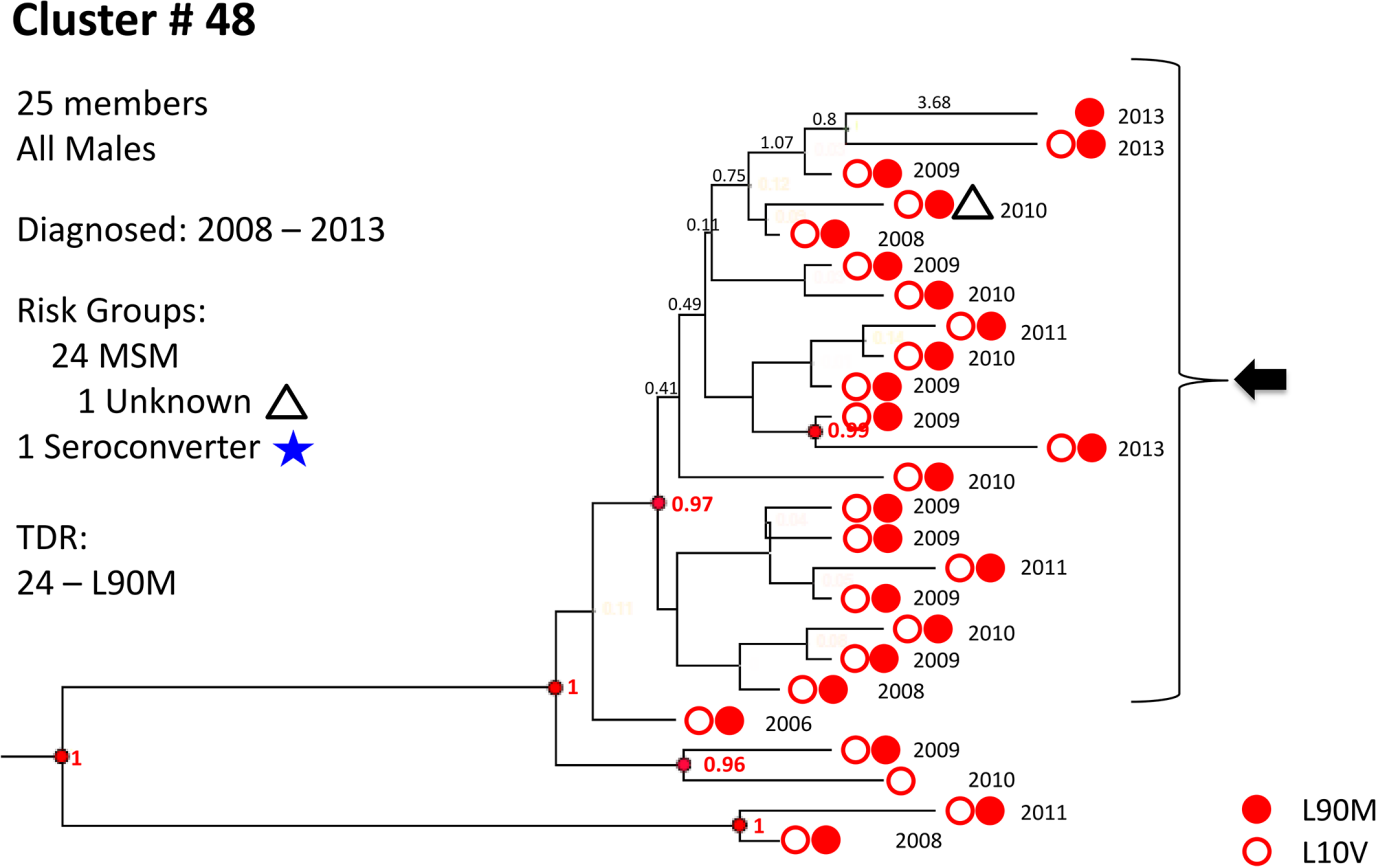
Cluster # 48. The first of three examples of large subtype-B clusters embedded in Fig 4. Cluster 48 is composed of 25 males, 24 of them MSM, diagnosed and genotyped between 2008 and 2013. All but one harbored the protease mutations L90M and L10V; the remaining one had only L10V. The group contains one seroconverter who was diagnosed in 2008. 20 members had a posterior probability of recent common ancestor >0.99 and had short branches. Year of genotyping is indicated as well as branch lengths in years and selected resistance related mutations. Posterior probabilities ≥0.99 are indicated in red. Blue asterisk–seroconverters; green triangles–heterosexual males; green circles–heterosexual females; white triangles–male, risk group unknown; yellow triangles–IVDU males; yellow circles–IVDU females; FSU, Former Soviet Union; Hetero, heterosexuals; IVDU, intravenous drug users; MSM, men who have sex with men.

**Fig 6:**
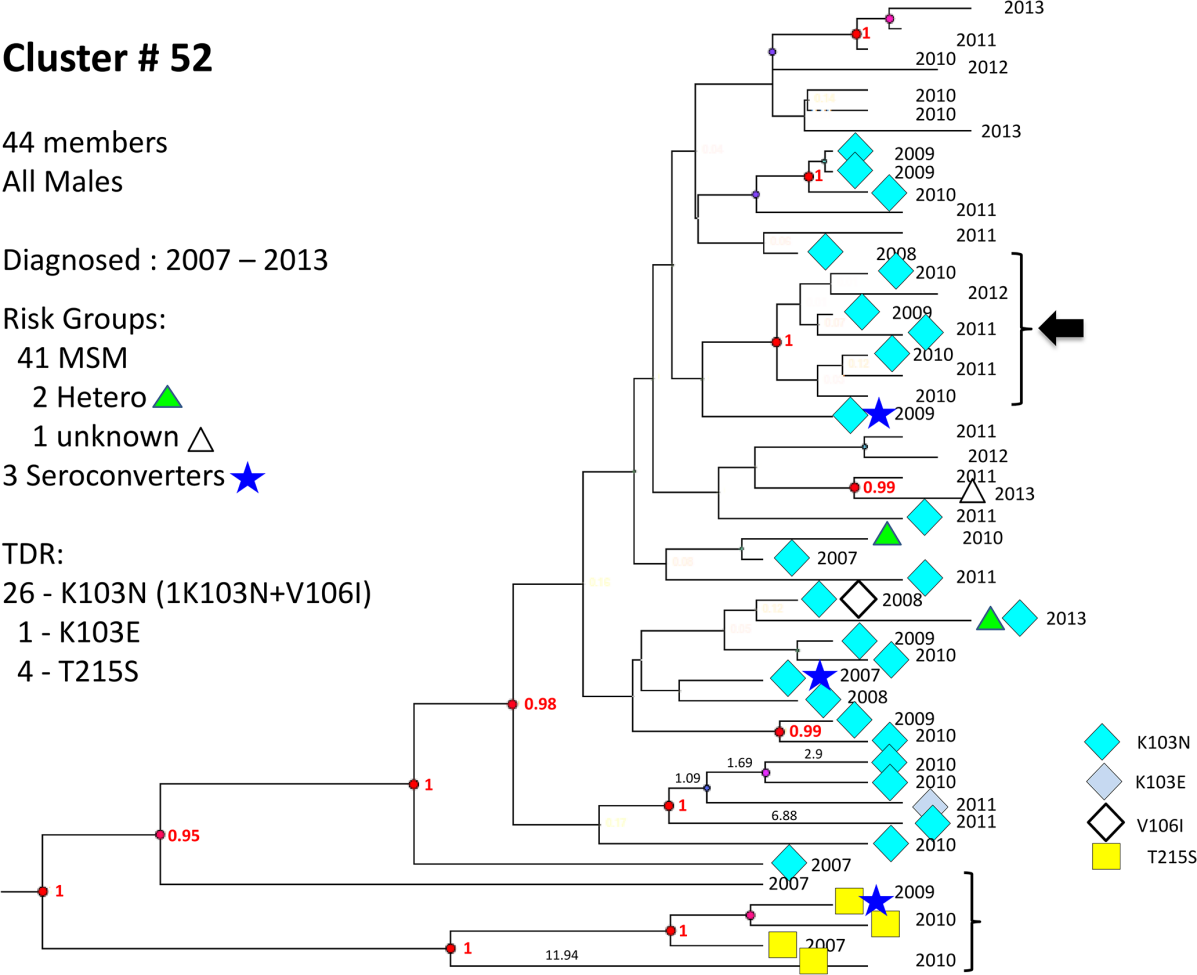
Cluster # 52. Cluster 52 is composed of 48 males, diagnosed and genotyped between 2007 and 2013. 26 harbored K103N, one K103E, and 4 had T215S. The group contains 3 seroconverters who were diagnosed 3 years apart (in 2007 and 2009). A transmission network of 7 members is noted (arrow). Notations as in Fig 5.

**Fig 7:**
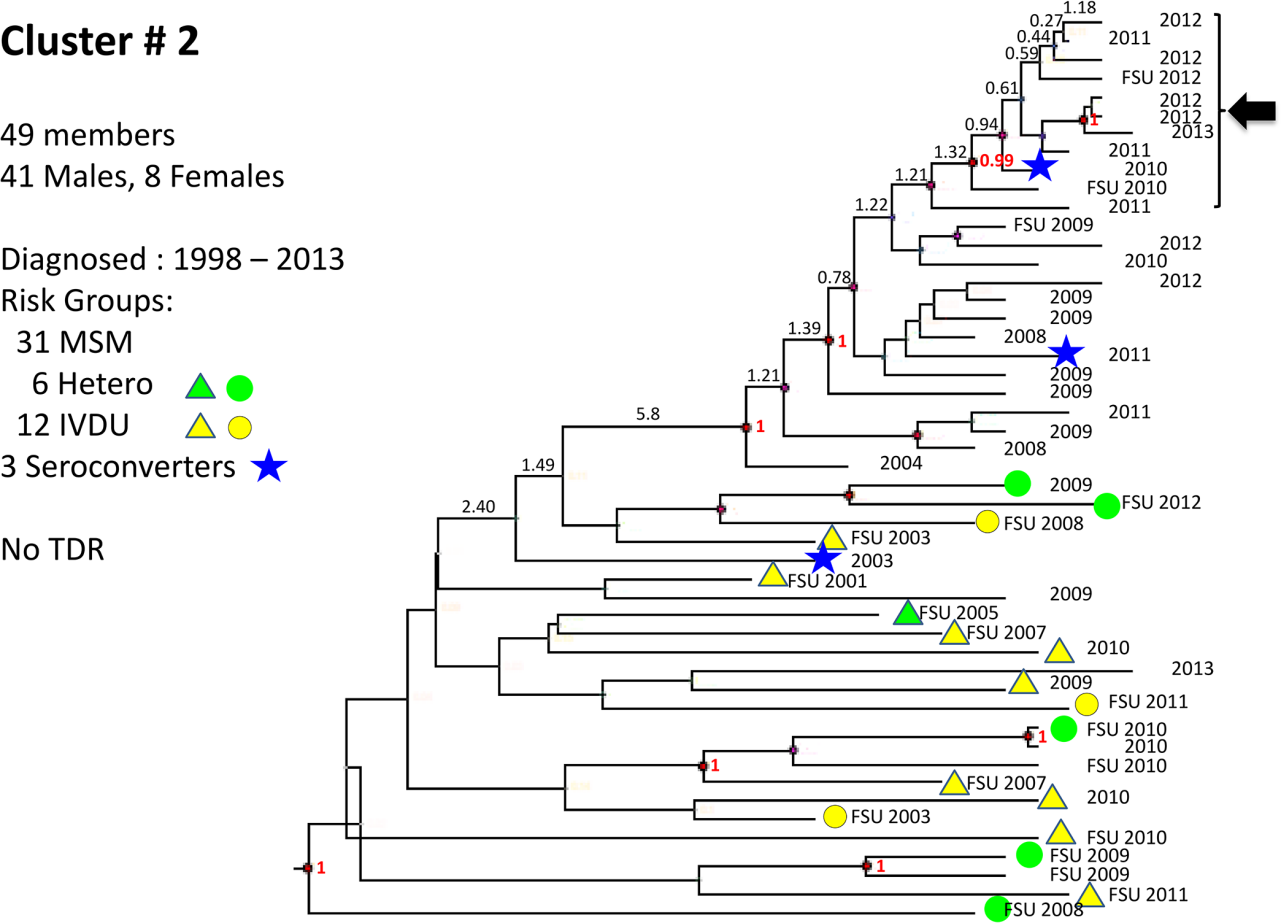
Cluster # 2. Cluster 2 is composed of 41 males and 8 females, diagnosed between 1998 and 2013 and genotyped between 2007 and 2013. The group contains 3 seroconverters who were diagnosed 7 years apart (in 2003, 2010 and 2011). A transmission network of 10 members is noted (arrow). Notations as in Fig 5.

In contrast to the lack of large clusters in subtypes A and C viruses, analysis of subtype-B viruses demonstrated a number of large clusters with common ancestors having posterior probability ≥ 0.95: 82% of all subtype-B sequences were present in lineages with high probability (≥0.95) of a common ancestor (Fig 4). BEAST analysis revealed that large clusters of subtype-B infections had estimated most recent common ancestors dating to 2000–2002 (Fig 4) suggesting that the viruses in these groups had been circulating for years; several clusters had TMRCA dating into the 1980’s (Fig 4) including as early as 1981, approximating the arrival time of some of the initial cases of HIV in Israel [8]. Sequences from seroconverters (N = 37) were present in the subtype B dataset, and several groups included seroconverters that were identified years apart; as shown (Figs 5–7, asterisks), nearly identical viruses were documented in individuals with seroconversion events occurring more than 9 years apart, clearly demonstrating prolonged persistence of individual lineages.

Within the large B clusters there were smaller clusters with posterior probabilities >0.95 and relatively short branch lengths, suggesting the presence of transmission networks with a recent common ancestor (Fig 4). Analysis of these transmission networks (S1 Table) revealed that individual clusters had members with ages that were significantly younger (median age of youngest member of network A in cluster 1 was 22.3 years, compared to overall subtype-B infected population, 34.3 years; *p* = 0.002,) or older (median age of the oldest network in cluster 33, network B, was 49.5 years, older than the overall subtype-B infected population; *p* = 0.02), suggesting some networks had distinct age characteristics (S1 Table).

The majority of subtype-B infections were present in Israeli-born individuals, but a substantial number (N = 100, 13%, Table 1) were diagnosed in FSU immigrants. Bayesian analysis revealed that 89% of these B-infected FSU immigrants were present in large clusters that included Israeli-born individuals, suggesting that these transmissions occurred within Israel (*e*.*g*. Fig 7).

Although the majority of subtype-B infected reported MSM exposure, fine-structure analysis using demographic information to annotate BEAST topologies revealed heterogeneity among individual clusters. Some clusters were exclusively composed of MSM, while others were heterogeneous and included IVDU and individuals reporting heterosexual transmission, often with significant excess of IVDU over the prevalence of IVDU in the entire subtype B population (*p*<0.00008). Women composed 5% of the groups, which was not significantly different from the overall presence of subtype B in women (5.3%; *p* = 0.8). Typically, women appeared in clusters along with males reporting heterosexual or IVDU as HIV transmission risk factors.

The number of B sequences analyzed (N = 770) was substantial, but was not the reason why so many were present in groups. Indeed, multiple replicate analyses using random samples of smaller numbers of sequences (N = 350) also yielded topologies with large sequence clusters, and with relatively few common ancestors having posterior probability ≥ 0.95. In addition, increasing the number of reference sequences did not divide the clusters (data not shown). These data indicate that the current HIV B epidemic in Israel has a limited number of ancestral HIV B sequences.

### Recombinant Strains

Co-circulation of distinct subtypes in geographically delimited areas provides the potential for inter-subtype recombination. We investigated whether inter-subtype recombination was detectable by measuring correlation of protease and RT subtype assignment in each genotype. We noted four examples of protease RT subtype discordance. We analyzed these genotypes using SIMPLOT approaches to determine the nucleotide positions of potential crossover events and compare them to known circulating recombinant forms [15,16]. As shown in S4 Fig, several potential recombinants were identified that have not been previously reported, suggesting co-circulation of these subtypes can result in inter-subtype recombination. Detection of potential new recombinant viruses is consistent with transmissions across traditional groups. Two isolates (numbers 121 and 251, S4 Fig) from male immigrants reporting heterosexual and MSM transmission category, respectively, originating in Ethiopia and FSU in 1997 and 2001, respectively, had no similar recombinant in the Los Alamos database. A few additional samples clustered with CRF 03-AB and CRF 62-BC (S4 Fig, isolates 1102 and 1819) and could not be unequivocally determined to have occurred in Israel.

### Transmitted Drug Resistance

Overall, TDR mutations were found in 157/1,427 drug-naïve patients (11%). TDR rates were significantly higher in subtype B (13%) than in A (6%) and C (9%; *p* = 0.004 and 0.03 for B *vs*. A and B *vs*. C, respectively; *p* = 0.2 for A *vs*. C). Overall rates of TDR to PI and NRTI (4% and 3%, for PI and NRTI, respectively; 1%-5% across the different HIV transmission categories) were lower than TDR to NNRTI (6% total, 4%-7% across groups; *p*<0.02 and *p*<0.0001 for NNRTI *vs*. PI and NRTI, respectively). Eleven patients (0.7%) had resistance to two classes of drugs and four patients (0.3%) had resistance to three classes (Table 2). TDR rates were significantly higher in individuals reporting MSM (14.5%) than among those reporting hetero (9.3%) and IVDU (4.7%) risk factors (*p* = 0.008 and 0.0006 for MSM *vs*. hetero and IDVU, respectively). As a substantial number (73%) of individuals reporting IVDU risk factors are infected with subtype A, this is consistent with an overall lower rate of TDR in subtype A.

**Table 2:** Transmitted drug resistance (TDR).

		A (n = 232)		B (n = 770)		C (n = 425)		Total (N = 1427)		*P*		
		N	%	N	%	N	%	N	%	A *vs*. B	B *vs*. C	A *vs*. C
**PI**	**L24I**			**1**	**0.10%**			**1**	**0.10%**			** **
	**V32I**	**1**	**0.40%**					**1**	**0.10%**			** **
	**M46I**	**3**	**1.30%**	**7**	**1.00%**	**7**	**1.60%**	**17**	**1.20%**			** **
	**F53L**			**3**	**0.40%**	**2**	**0.50%**	**5**	**0.40%**			** **
	**I54V**			**3**	**0.40%**	**2**	**0.50%**	**5**	**0.40%**			** **
	**V82A**			**4**	**0.50%**	**2**	**0.50%**	**6**	**0.40%**			** **
	**G73S**			**1**	**0.10%**	**1**	**0.20%**	**2**	**0.10%**			** **
	**N88D**			**1**	**0.10%**	**1**	**0.20%**	**2**	**0.10%**			** **
	**L90M**	** **	** **	**31**	**4.30%**	**3**	**0.80%**	**34**	**2.40%**	**>0.002**	**>0.001**	**0.2**
**NRTI**	**M41L**	**2**	**0.90%**	**7**	**1.00%**	**1**	**0.20%**	**10**	**0.70%**			** **
	**K65R**					**1**	**0.20%**	**1**	**0.10%**			** **
	**D67N,G,E**	**2**	**0.90%**	**4**	**0.50%**	**2**	**0.50%**	**8**	**0.60%**			** **
	**T69D**			**1**	**0.10%**			**1**	**0.10%**			** **
	**K70 E**			**1**	**0.10%**			**1**	**0.10%**			** **
	**L74V**			**1**	**0.10%**			**1**	**0.10%**			** **
	**F77L**	**2**	**0.90%**	**1**	**0.10%**	**1**	**0.20%**	**4**	**0.30%**			** **
	**F116Y**					**1**	**0.20%**	**1**	**0.10%**			** **
	**M184I/V**	**2**	**0.90%**	**3**	**0.40%**	**5**	**1.20%**	**10**	**0.70%**			** **
	**L210W**	**1**	**0.40%**	**1**	**0.10%**		**%**	**2**	**0.10%**			** **
	**T215Y**	**1**	**0.40%**	**16**	**2.80%**	**6**	**1.40%**	**23**	**1.60%**	**0.1**	**0.4**	**0.2 **
	**K219E,N,Q,R**	** **	** **	**4**	**0.50%**	**2**	**0.50%**	**6**	**0.40%**	** **	** **	** **
**NNRTI**	**K101E, P**	**1**	**0.40%**			**6**	**1.40%**	**7**	**0.50%**			** **
	**K103N**	**8**	**3.40%**	**48**	**6.20%**	**9**	**2.10%**	**65**	**4.60%**	**0.1**	**0.001**	**0.3**
	**V106M**					**1**	**0.20%**	**1**	**0.10%**			** **
	**V179F**	**1**	**0.40%**					**1**	**0.10%**			** **
	**Y181C, V**			**3**	**0.40%**	**2**	**0.50%**	**5**	**0.40%**			** **
	**Y188H**			**1**	**0.10%**			**1**	**0.10%**			** **
	**G190A**					**3**	**0.80%**	**3**	**0.20%**			** **
	**P225H**	** **	** **	**1**	**0.10%**	** **	**%**	**1**	**0.10%**	** **	** **	** **

TDR mutations in drug-naïve patients found in different subtypes. TDR were identified according to Bennett et al. [17]. Only mutations that appeared at least once are listed. TDR were found significantly more among MSM, and some transmission networks included predominantly individuals with TDR, but somewhat surprisingly, no significant difference in TDR frequency was found in clustered individuals (i.e., within identifiable lineages) versus non-clustered. Two mutations, protease L90M and RT K103N/S, were present in clusters of MSM (Fig 3). T215FY/D/E/N/S (1.7%) and M184V (0.8%) were similarly distributed among clustered and non-clustered patients.

No significant differences in overall TDR frequency were detected in clusters compared with non-clustered B and C viruses, but more TDR was found in clustered than non-clustered A infections (*p* = 0.02). Two mutations, protease L90M and RT K103N/S, were present in clusters of MSM (*p* = 0.02 and *p*<0.0005 for clustered *vs*. non-clustered L90M and K103N/S, respectively). The frequency of these two mutations was significantly higher in subtype B patients (Table 2, Figs 5 and 6). Additional TDR details are provided in S2 Table.

## Discussion

Understanding the spread of HIV infection in risk-group populations is a public health imperative to controlling the epidemic. Assessing behavioral patterns and infection-spread hotspots typically relies on active surveillance and contact tracing. Existing resources and difficulties in appropriate population sampling [1] limit standard epidemiologic approaches, and HIV molecular epidemiology has been used as an adjunct. HIV genotyping at the time of diagnosis is currently recommended (http://aidsinfo.nih.gov/guidelines) to identify transmitted drug resistance and inform initial therapeutic decisions. As a consequence of routine HIV care, therefore, a powerful approach to characterize HIV spread is readily available [6]. Routine sequencing data can be subjected to extensive phylogenetic reconstructions that can identify closely-related viral sequences and determine whether lineages may have a common ancestry. Obviously, these methods cannot identify transmitting partners, but they generate useful information regarding the ongoing spread of HIV and can guide intervention steps.

We analyzed an extensive pre-therapy sequence dataset (N = 1,427). The majority of HIV infections continue to reflect initial founder characteristics: B in Israeli-born MSM, C in heterosexuals of Ethiopian origin and A in FSU immigrants. Cross-ethnic transmissions were also present, with Subtype-A and C infections in Israeli-born and subtype-B and C infections in FSU immigrants (Table 1), but spread of subtype A and B into the Ethiopian-origin population remained very limited. Phylogenetic analysis suggested that cross-ethnic spread was not due to single events but to multiple independent introductions, with a prevalent role of MSM in transmissions (Fig 2 and S3 Fig). There were few clusters of subtype A or C infections, and several of the ones we did detect were composed of only 2 or 3 individuals; clusters of such small size provide little if any epidemiologic information. A limited number of clusters with ≥4 individuals were detected in subtype A and C, and were almost invariably composed of individuals reporting MSM as a transmission category (Figs 2 and 3).

The B-dataset (N = 770) represents approximately 50% of all diagnosed subtype-B infections; the A- and C-datasets represent *c*. 22% and 18% of all subtype-A and subtype-C infections, respectively. Multiple discrete groups with high posterior probabilities and short branch lengths, possibly representing distinct transmission networks, included members of all HIV transmission categories, but MSM was most frequent; such groups may be critical to ongoing spread of HIV in Israel, including cross-ethnic transmissions. Examples are detailed in Figs 5–7 and S1 Table. Analysis of subtype-A and-C infections did not identify large non-MSM clusters, although a substantial number of smaller groups were identified. For subtype A, a group of 7 and a group of 12 were identified, all but one reporting MSM exposure. The absence of substantial grouping (at least until recently) in non-MSM subtype A and in subtype C is explained in part by the fact that subtype A and subtype C viruses derived initially from a large number of independent introductions. Indeed, about half of C infections and a large fraction of A occurred in the countries of origin in Africa and the FSU, respectively; even though immigrants from these geographic locations represent a relatively small group, they still brought genetically diverse viral strains. The reference strains used for this study from the Los Alamos reference set typically included strains with the earliest isolation dates, and were diverse and unrelated to sequences reported here, illustrating the difficulty in obtaining an accurate estimate of the number of independent introductions of A and C viruses. In contrast, it is likely that only a few introductions of subtype B occurred initially (Fig 4). In addition, subtype A and subtype C spread within Israel in a much more sporadic fashion (with the exception of infections involving MSM), diminishing local formation of clusters. Indeed, the overall doubling time in IVDU, 9.8 years, and ethnic Ethiopians, 12.6 years, was longer than that of MSM, 5.5 years (data not shown), despite continued low-level immigration of infected individuals from FSU and Ethiopia. Interestingly, in a recent phylogenetic analysis in British Columbia, Canada, IVDU were significantly more likely to appear in a cluster than MSM [32].

We analyzed a 918 nucleotide region of HIV protease-RT. A number of inter-subtype recombinants were found and four of them have been described (see Results). It is possible that additional recombinants may exist but remain undetected because sequences of entire genomes were not analyzed.

Transmitted drug resistance was present in all subtypes, but most common in subtype B (Table 2). BEAST analysis revealed that some lineages of resistance mutations had recent common ancestors with posterior probability ≥0.95 (Figs 5–7), but many were distinct, suggesting transmitted drug resistance is a pervasive issue not confined to a limited number of transmission groups.

Previous phylogenetic analyses have yielded insights into the origins and early spread of HIV [33–36]. In Central Europe, FSU, and Pakistan, intermixing between IVDU and MSM was responsible for a “bridging” effect, spreading HIV to women and subsequently children [37]. In the present study, ongoing cross-ethnic transmission in Israel was detected largely through groups with MSM, but not IVDU, suggesting that MSM represent a substantial source of “bridging”. In Israel, recent observations of new clusters of HIV, including new IVDU transmission networks, have been reported using standard epidemiologic approaches, in particular since the introduction of new synthetic drugs [38]. Comprehensive molecular epidemiology methods to track HIV spread such as those described here represent useful adjuncts to investigate ongoing and emerging transmission networks. Similarly, as new approaches to prevent transmission are implemented, the combination of epidemiologic, phylogenetic and Bayesian analyses to characterize the size and composition of transmission clusters will provide useful quantifiable outcome metrics.

Our study had several limitations. Our sample size was substantial, but was not strictly random. As previously described [6], MSM access healthcare more frequently than other groups. It is likely that MSM are sampled more extensively than other groups. The patients studied here were exclusively drug-naïve, and their genotypes were not compared to those of treatment-experienced individuals; we did identify transmission of drug resistant viruses, but we could not determine to what degree these transmissions were due to circulation of drug-resistant viruses in drug-naïve individuals without ongoing contribution of treatment-experienced individuals, as demonstrated by Hue et al. [39]. Additional analyses are necessary to evaluate the contribution of resistant strains circulating among drug naïve individuals. Also missing in this analysis is rigorous determination of HIV infection time, to facilitate calculation of incidence rates and to better define lineage evolution histories. New approaches to uniformly estimate the duration of infection are presently being investigated.

The finding of multiple distinct lineages, some with drug resistance, demonstrates that complex transmission dynamics can be delineated using detailed genetic analyses. Reconstructed phylogenetic trees of subtype-B sequences versus subtype-A and-C sequences reflect differences in transmission dynamics linked to HIV transmission categories. Such data provide a baseline to track epidemic trends and will be useful in informing and quantifying efforts to reduce HIV transmission.

## Supporting Information

S1 Fig Demographic characteristics of patients included in the study. HIV subtypes, HIV Transsmission categories, and country of origin of patients included in the study. Patients are stratified according to subtype (large circle) or HIV Transmission Categories (smaller circles). Et–Ethiopia; FSU–Former Soviet Union; Hetero–heterosexuals; IVDU–intravenous drug users; MSM–men who have sex with men.
(PDF)

S2 Fig Trends of the HIV epidemic in Israel. Upper panel shows risk-group constitution of newly diagnosed patients between 1986 and 2012. A moving three-year average was recalculated each year. The lower panel shows changes in numbers of newly diagnosed individuals belonging to different risk groups between 2005 and 2013. Based on epidemiological data provided by the Department of Tuberculosis and AIDS of Israel Ministry of Health [8]. Note: All Ethiopian immigrants are tested for HIV upon arrival to Israel. The decrease in newly-diagnosed Ethiopian-origin patients in last years is mainly due to a decline in the number of new immigrants since 2008. Since 2007, MSM is the largest risk-group among newly diagnosed. Et–Ethiopia; Hetero–heterosexuals; IVDU–intravenous drug users; MSM–men who have sex with men.
(PDF)

S3 Fig Phylogenetic analysis of transmission groups (maximum likelihood trees). Phylogenetic relations among HIV sequences sampled from IVDU, Hetero, and MSM calculated by maximum likelihood based on the GTR+G nucleotide substitution model [20]. Reference sequences (N = 138; 61 subtype A, 47 subtype C, and 30 subtype B) from the Los Alamos database were added to each group. The tree with the highest log likelihood is shown in each case. The tree is drawn to scale, with branch lengths measured in the number of substitutions per site. All ambiguous positions were removed in comparing sequence pairs. There were a total of 791 positions in the final dataset. Branch reproducibility was assessed with 1,000 bootstrap replicates using MEGA6 [23]. Among the 109 subtype-A IVDU patients from FSU, only 8 (7.3%) were in clusters (all couples), reflecting the wide dispersion of subtype-A HIV lineages in the country of origin. In contrast, 20 of 36 IVDU patients with subtype B (56%) were clustered, including a group of 12. This indicates penetrations of subtype-B virus into FSU IVDU occurring in Israel. Indeed, the 12 closely-related IVDU B-sequences (bootstrap support >80 and posterior >99) grouped with 27 Israeli-born MSM and others, implicating risk-group intermixing (Figs 3 and 7). Accession numbers of the reference sequences are provided as “S1 Text”. Thick lines represent branches with bootstrap support >80; blue triangles, born in Israel; red squares, born in FSU; white squares, born elsewhere; green diamonds, Ethiopian origin; A, subtype A; B, subtype B; C, subtype C; FSU, Former Soviet Union; Hetero, heterosexuals; IVDU, intravenous drug users; MSM, men who have sex with men.
(PDF)

S4 Fig Recombinants among A, B and C. Four examples of recombinant viruses found among HIV infected patients. Recombinants between established subtypes were identified using the REGA Subtyping Tool [15] and SimPlot [16].
(PDF)

S1 Table Transmission networks. Characteristics of potential transmission networks embedded in larger clusters.
(DOCX)

S2 Table Resistance related mutations. Accessory mutations and polymorphisms listed by subtype.
(DOCX)

S1 Text Accession numbers. Accession numbers of sequences of Israeli patients and reference sequences from the Los Alamos database used in this study.
(DOCX)
