# Supplementary material for: A Population-Structured HIV Epidemic in Israel: Roles of Risk and Ethnicity
Source: PLoS One. 2015 Aug 24;10(8):e0135061. doi: 10.1371/journal.pone.0135061 (PMC4547742; doi:10.1371/journal.pone.0135061)
Supplement: S1 Fig — HIV subtypes, HIV Transsmission categories, and country of origin of patients included in the study. Patients are stratified according to subtype (large circle) or HIV Transmission Categories (smaller circles). Et–Ethiopia; FSU–Former Soviet Union; Hetero–heterosexuals; IVDU–intravenous drug users; MSM–men who have sex with men. (PDF) [file pone.0135061.s001.pdf]

Total
N=1427

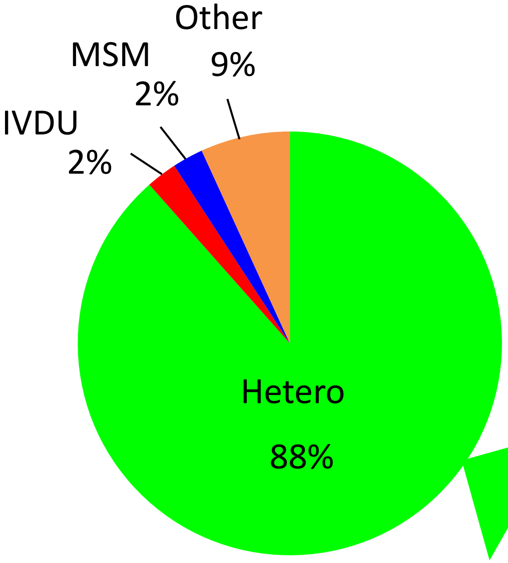

Origin	N	Percent
Ethiopian	377	89
FSU	10	2
Israeli	24	6
Other	14	3

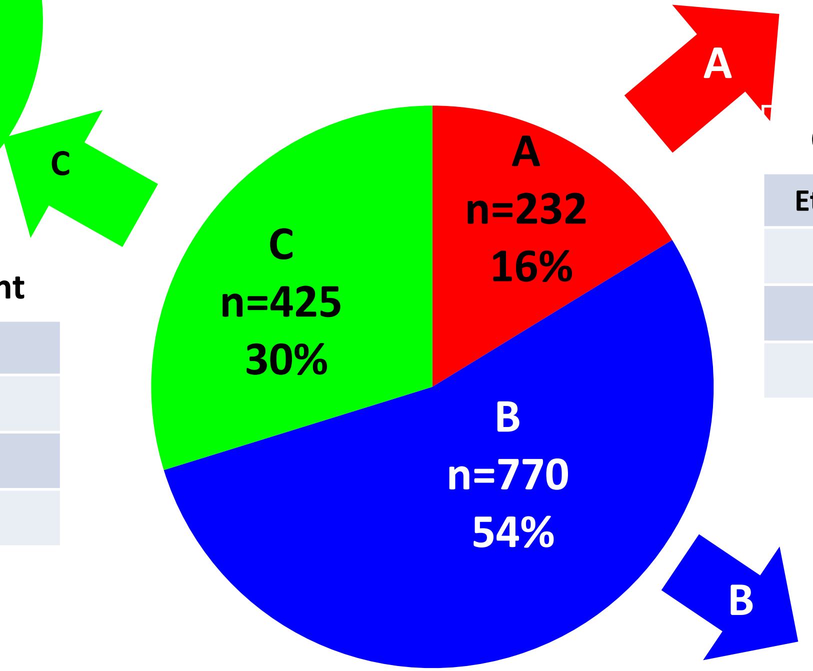

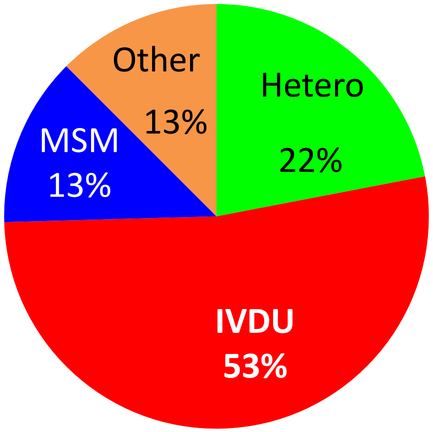

Origin	N	Percent
Ethiopian	3	1.3
FSU	165	71
Israeli	41	18
Other	23	10

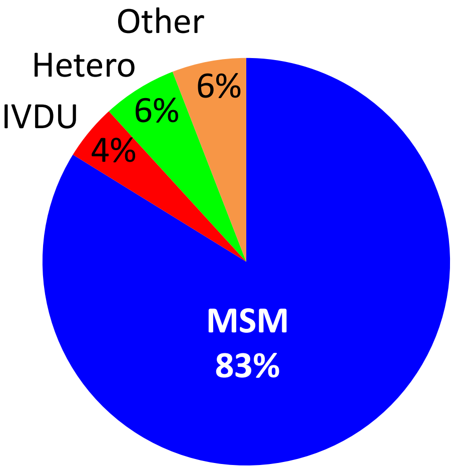

Origin	N	Percent
Ethiopian	2	0.3
FSU	100	13
Israeli	564	73
Other	104	14
