# Supplementary figures and images for: A Population-Structured HIV Epidemic in Israel: Roles of Risk and Ethnicity
Source: PLoS One. 2015 Aug 24;10(8):e0135061. doi: 10.1371/journal.pone.0135061 (PMC4547742; doi:10.1371/journal.pone.0135061)

Number of newly diagnosed patients

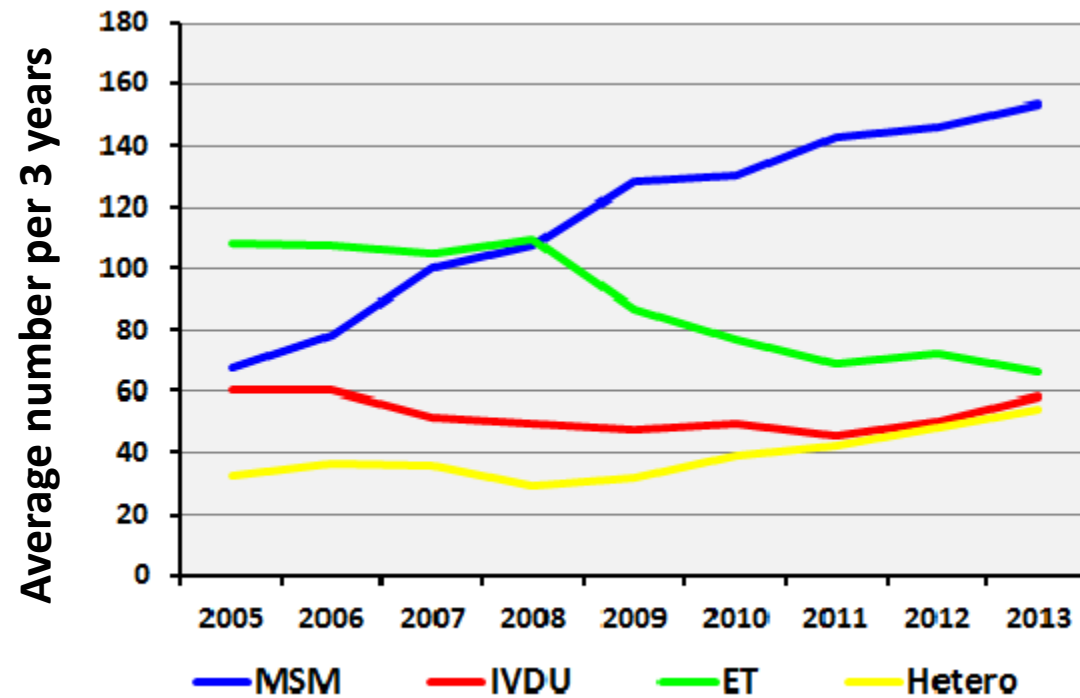

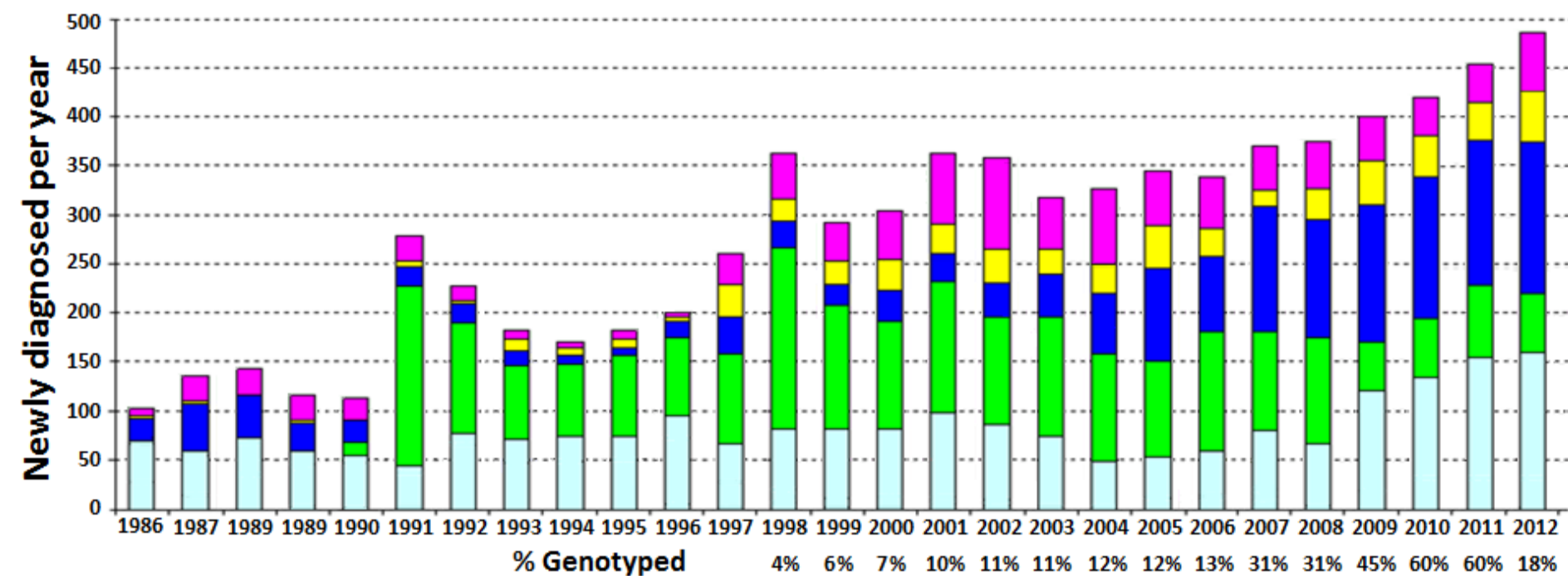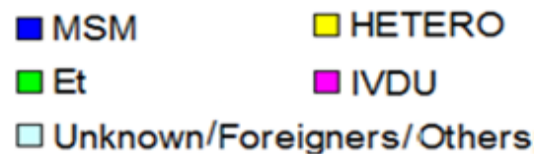

Supplement: S2 Fig — Upper panel shows risk-group constitution of newly diagnosed patients between 1986 and 2012. A moving three-year average was recalculated each year. The lower panel shows changes in numbers of newly diagnosed individuals belonging to different risk groups between 2005 and 2013. Based on epidemiological data provided by the Department of Tuberculosis and AIDS of Israel Ministry of Health [8]. Note: All Ethiopian immigrants are tested for HIV upon arrival to Israel. The decrease in newly-diagnosed Ethiopian-origin patients in last years is mainly due to a decline in the number of new immigrants since 2008. Since 2007, MSM is the largest risk-group among newly diagnosed. Et–Ethiopia; Hetero–heterosexuals; IVDU–intravenous drug users; MSM–men who have sex with men. (PDF) [file pone.0135061.s002.pdf]
