# Supplementary material for: A Population-Structured HIV Epidemic in Israel: Roles of Risk and Ethnicity
Source: PLoS One. 2015 Aug 24;10(8):e0135061. doi: 10.1371/journal.pone.0135061 (PMC4547742; doi:10.1371/journal.pone.0135061)
Supplement: S3 Fig — Phylogenetic relations among HIV sequences sampled from IVDU, Hetero, and MSM calculated by maximum likelihood based on the GTR+G nucleotide substitution model [20]. Reference sequences (N = 138; 61 subtype A, 47 subtype C, and 30 subtype B) from the Los Alamos database were added to each group. The tree with the highest log likelihood is shown in each case. The tree is drawn to scale, with branch lengths measured in the number of substitutions per site. All ambiguous positions were removed in comparing sequence pairs. There were a total of 791 positions in the final dataset. Branch reproducibility was assessed with 1,000 bootstrap replicates using MEGA6 [23]. Among the 109 subtype-A IVDU patients from FSU, only 8 (7.3%) were in clusters (all couples), reflecting the wide dispersion of subtype-A HIV lineages in the country of origin. In contrast, 20 of 36 IVDU patients with subtype B (56%) were clustered, including a group of 12. This indicates penetrations of subtype-B virus into FSU IVDU occurring in Israel. Indeed, the 12 closely-related IVDU B-sequences (bootstrap support >80 and posterior >99) grouped with 27 Israeli-born MSM and others, implicating risk-group intermixing (Figs 3 and 7). Accession numbers of the reference sequences are provided as “S1 Text”. Thick lines represent branches with bootstrap support >80; blue triangles, born in Israel; red squares, born in FSU; white squares, born elsewhere; green diamonds, Ethiopian origin; A, subtype A; B, subtype B; C, subtype C; FSU, Former Soviet Union; Hetero, heterosexuals; IVDU, intravenous drug users; MSM, men who have sex with men. (PDF) [file pone.0135061.s003.pdf]

Hetero

n=453

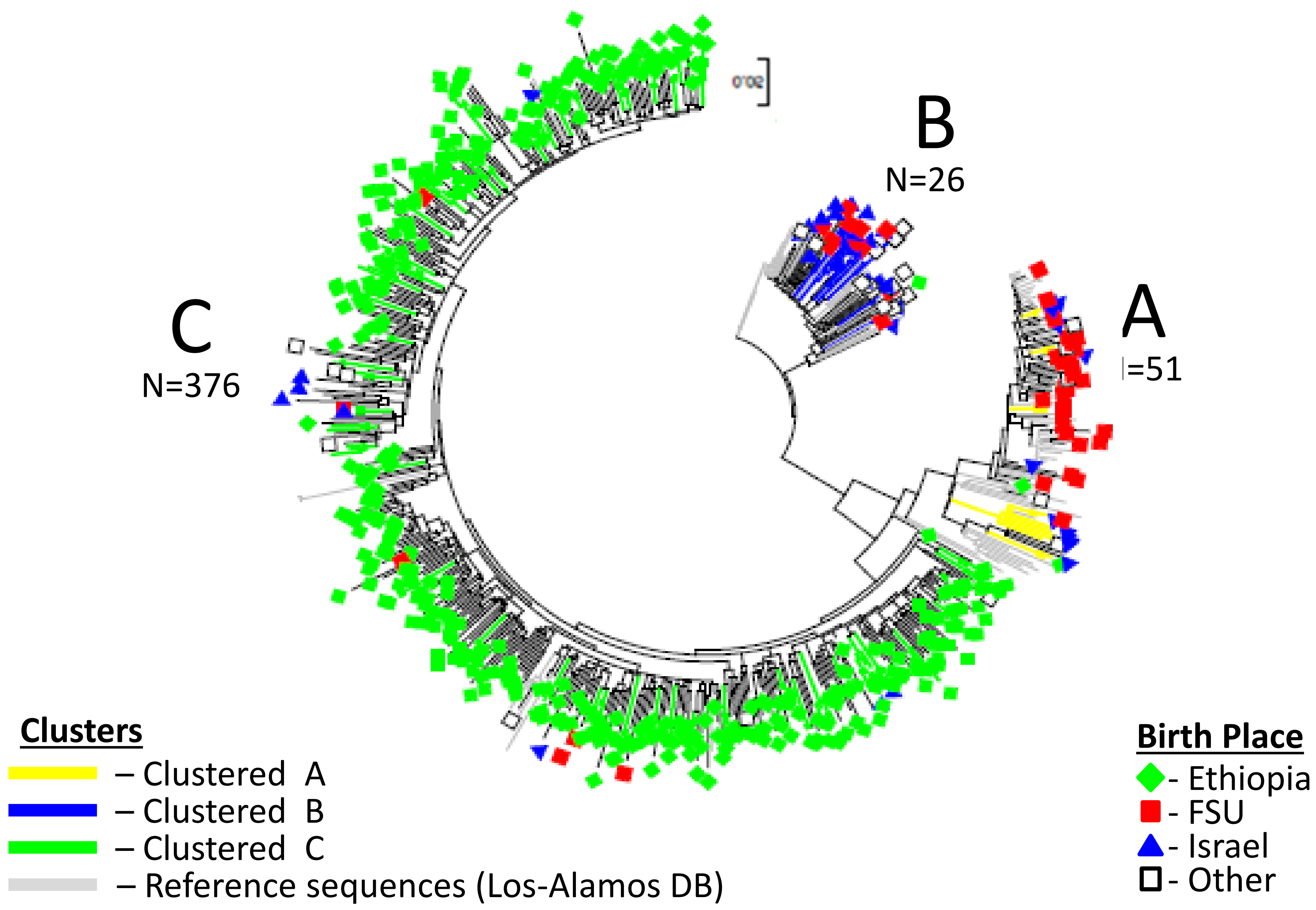

B
N=26
A
=51
C
N=376
Clusters
– Clustered A
– Clustered B
– Clustered C
– Reference sequences (Los-Alamos DB)
Birth Place
- Ethiopia
- FSU
- Israel
- Other

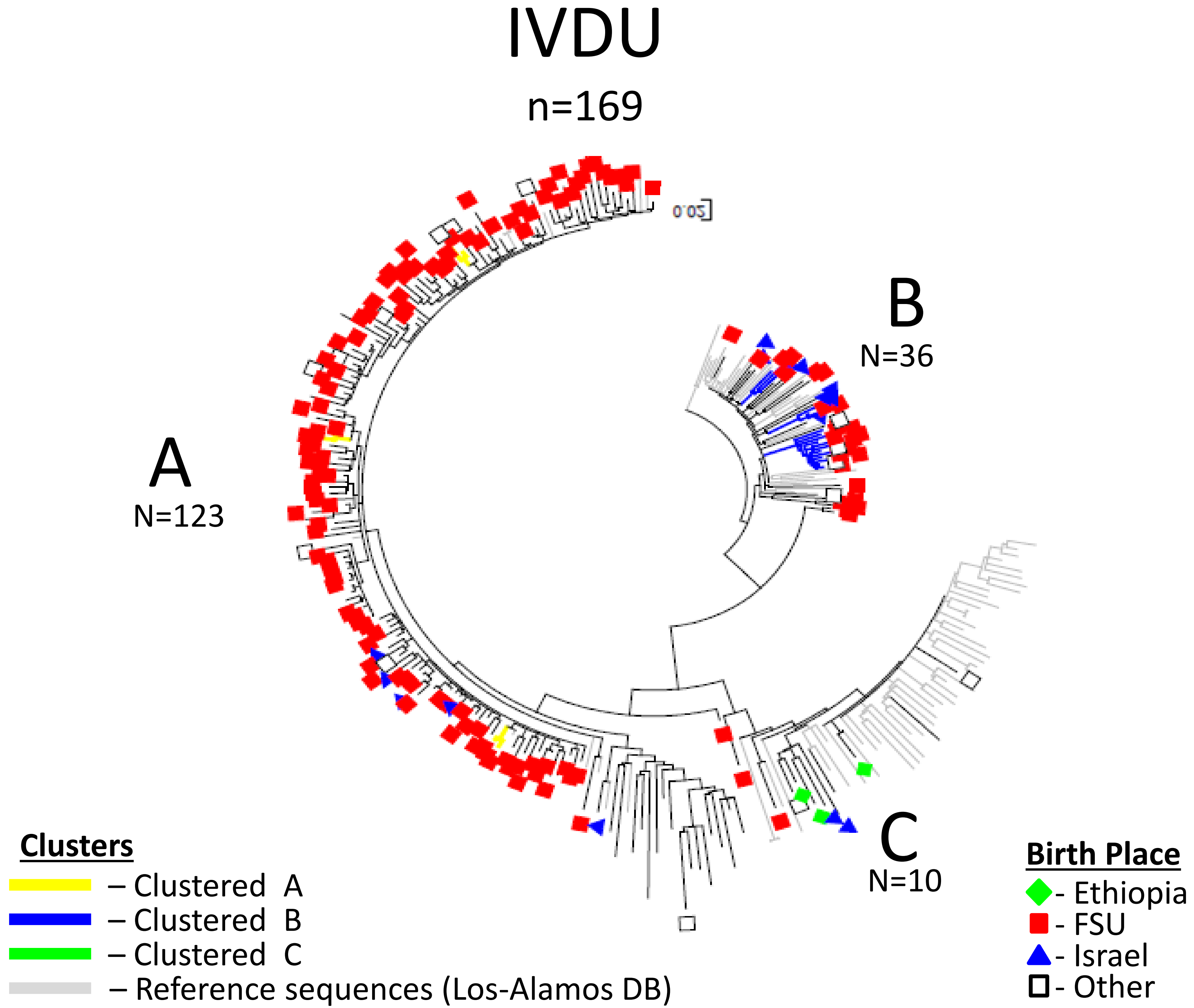
IVDU
n=169
A
N=123
B
N=36
C
N=10
Clusters
– Clustered A
– Clustered B
– Clustered C
– Reference sequences (Los-Alamos DB)
Birth Place
- Ethiopia
- FSU
- Israel
- Other

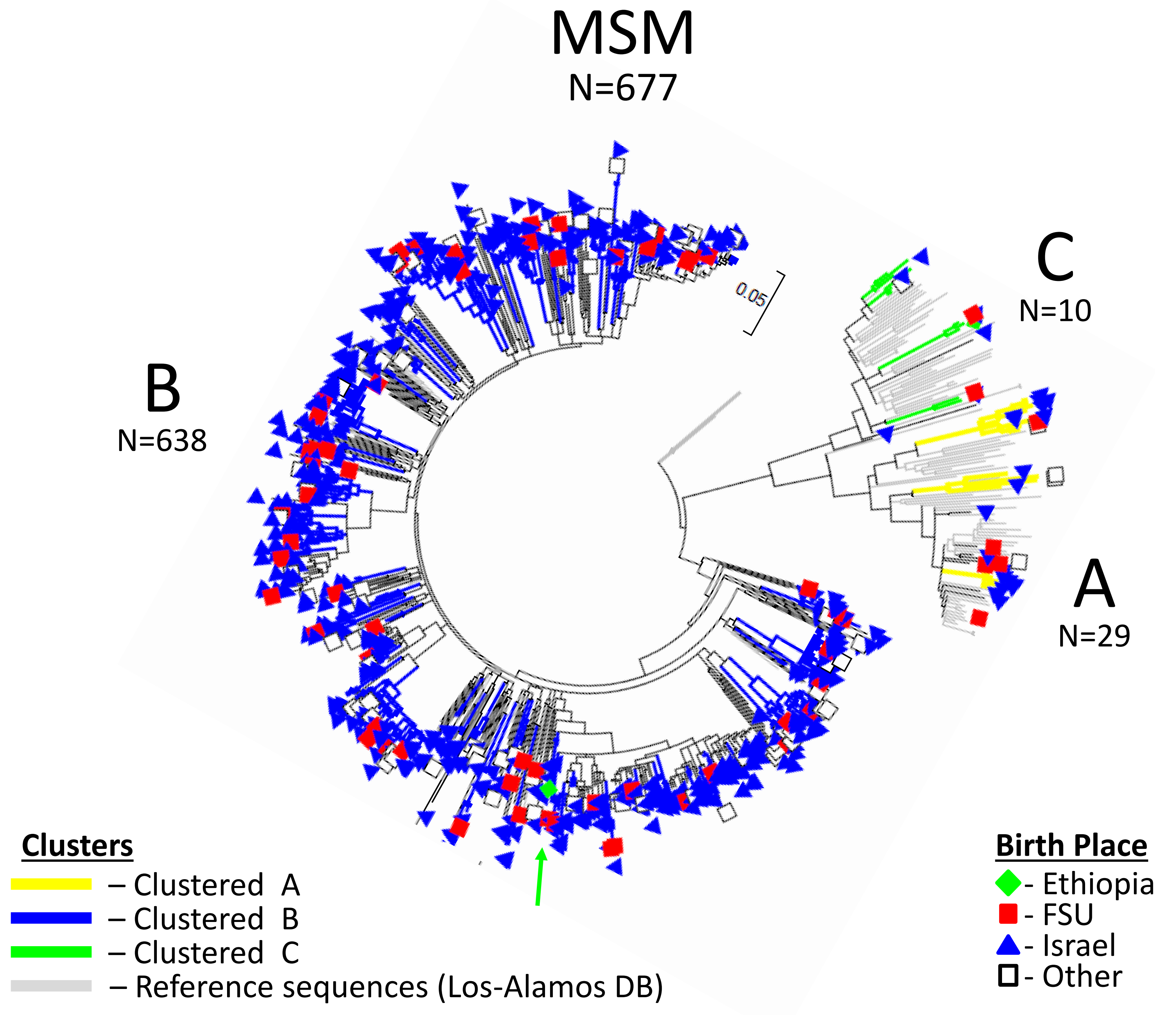
MSM
N=677
C
N=10
B
N=638
A
N=29
0.05
Clusters
– Clustered A
– Clustered B
– Clustered C
– Reference sequences (Los-Alamos DB)
Birth Place
- Ethiopia
- FSU
- Israel
- Other
