# Supplementary material for: A Population-Structured HIV Epidemic in Israel: Roles of Risk and Ethnicity
Source: PLoS One. 2015 Aug 24;10(8):e0135061. doi: 10.1371/journal.pone.0135061 (PMC4547742; doi:10.1371/journal.pone.0135061)

# 121_1997 C/A1

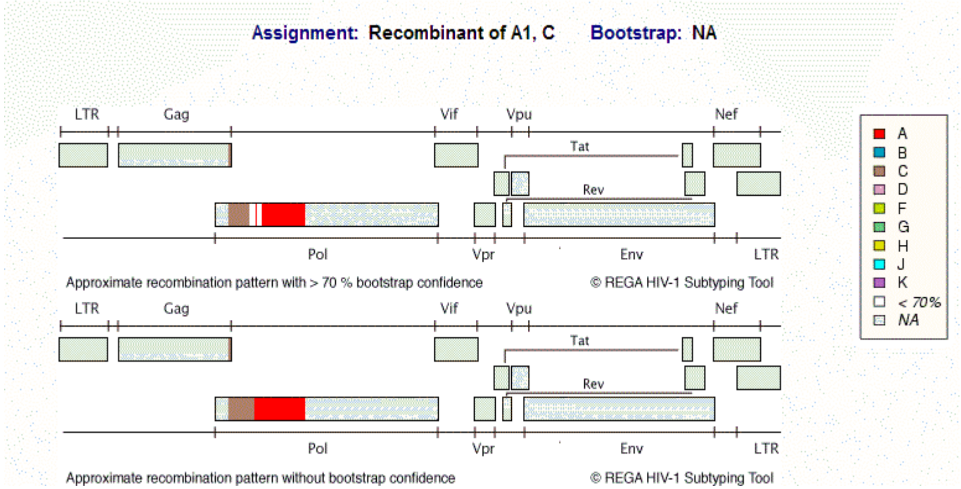
Assignment: Recombinant of A1, C
Bootstrap: NA
LTR
Gag
Vif
Vpu
Nef
Tat
Rev
Pol
Vpr
Env
LTR
Approximate recombination pattern with > 70 % bootstrap confidence
© REGA HIV-1 Subtyping Tool
Approximate recombination pattern without bootstrap confidence
© REGA HIV-1 Subtyping Tool
A
B
C
D
F
G
H
J
K
< 70%
NA

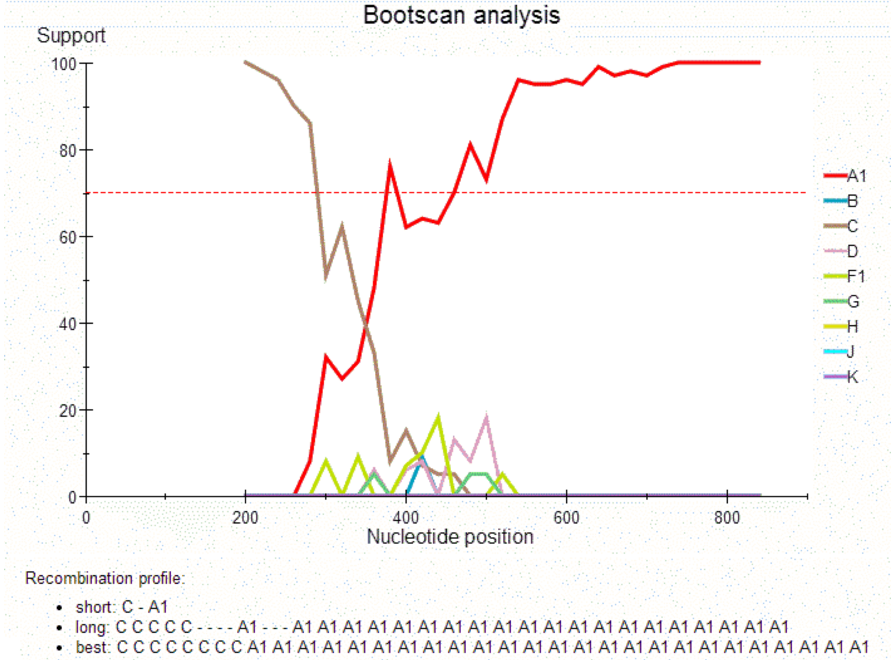
Bootscan analysis
Support
100
80
60
40
20
0
0
200
400
600
800
Nucleotide position
A1
B
C
D
F1
G
H
J
K
Recombination profile:
short: C - A1
long: C C C C C - - - - A1 - - - A1 A1 A1 A1 A1 A1 A1 A1 A1 A1 A1 A1 A1 A1 A1 A1 A1 A1 A1 A1
best: C C C C C C C C A1 A1 A1 A1 A1 A1 A1 A1 A1 A1 A1 A1 A1 A1 A1 A1 A1 A1 A1 A1 A1 A1 A1 A1 A1 A1 A1 A1

# 251_2001 A1/C

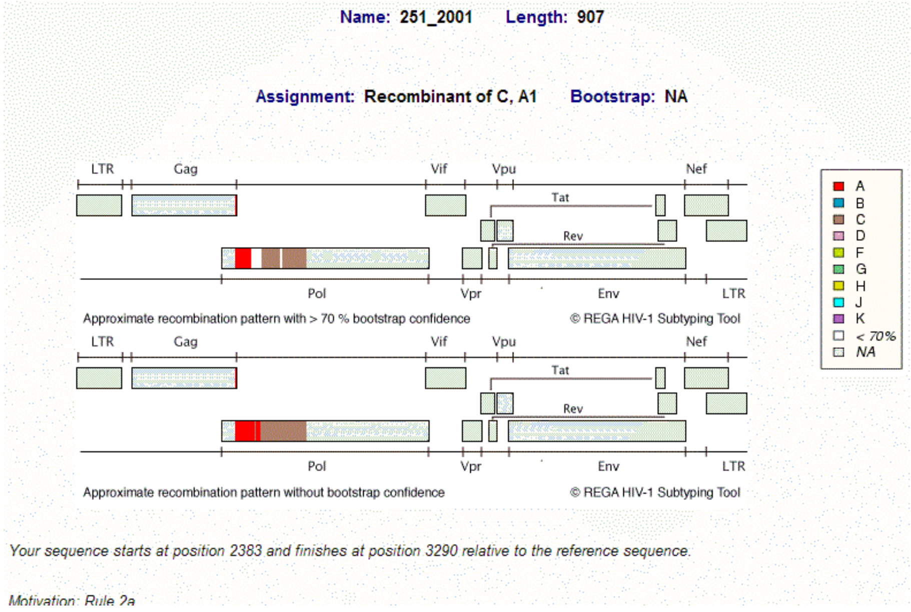
Name: 251_2001 Length: 907
Assignment: Recombinant of C, A1 Bootstrap: NA
LTR
Gag
Vif
Vpu
Nef
Tat
Rev
Pol
Vpr
Env
LTR
Approximate recombination pattern with > 70 % bootstrap confidence
© REGA HIV-1 Subtyping Tool
Approximate recombination pattern without bootstrap confidence
© REGA HIV-1 Subtyping Tool
A
B
C
D
F
G
H
J
K
< 70%
NA
Your sequence starts at position 2383 and finishes at position 3290 relative to the reference sequence.
Motivation: Rule 2a

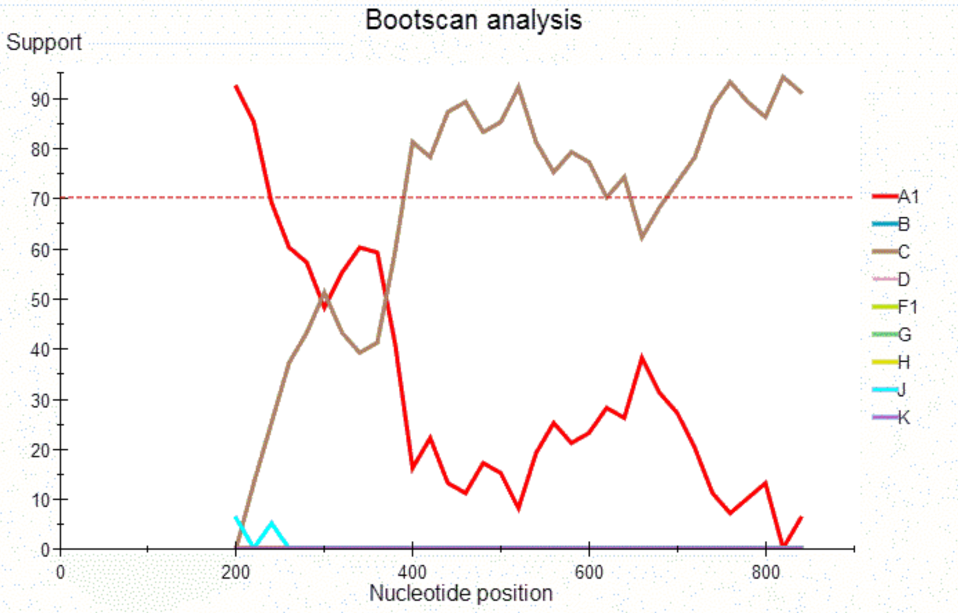
Bootscan analysis
Support
Nucleotide position
A1
B
C
D
F1
G
H
J
K

# 1102_1998_CRF 62-BC

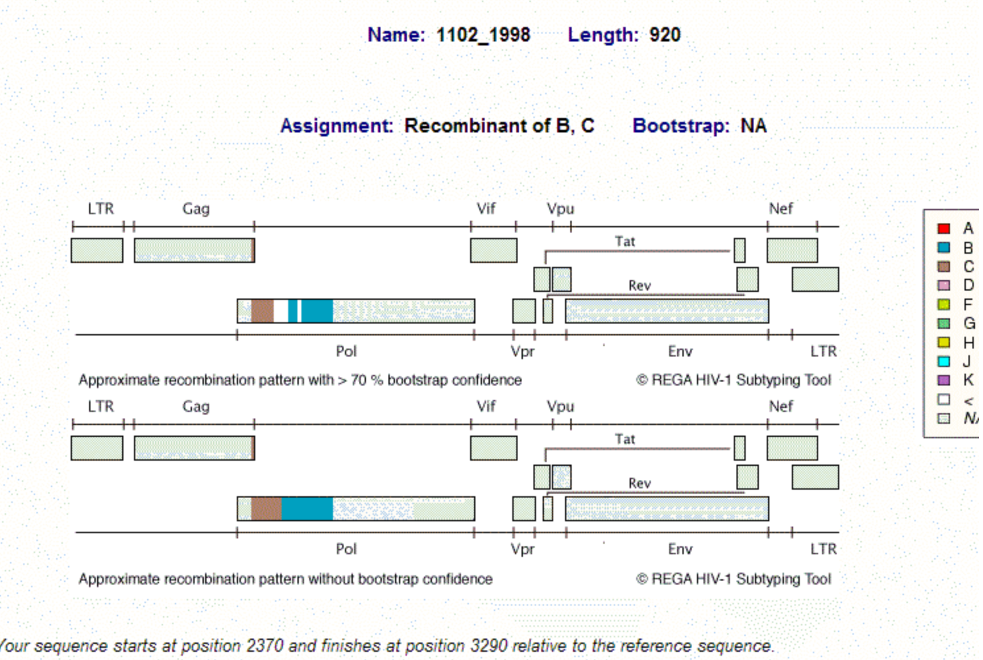


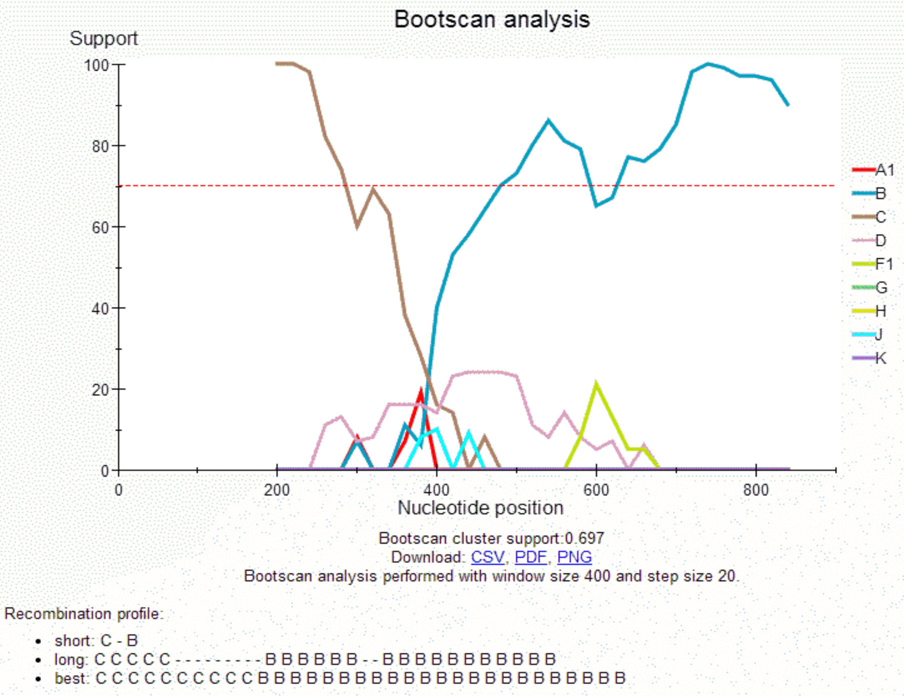

# 1819_2010_CRF-03_AB

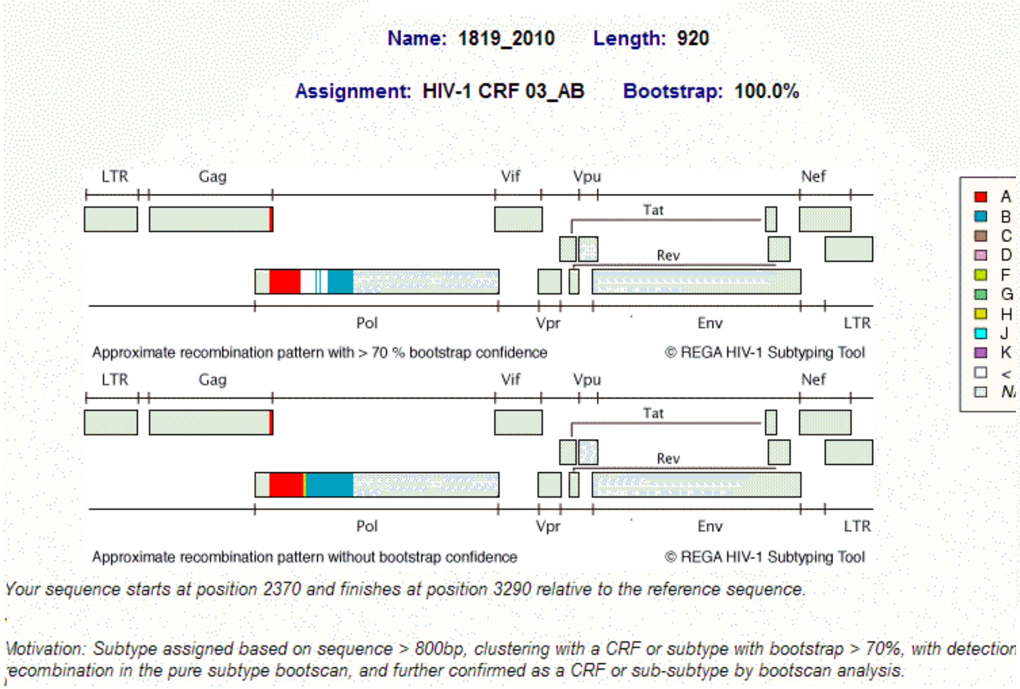


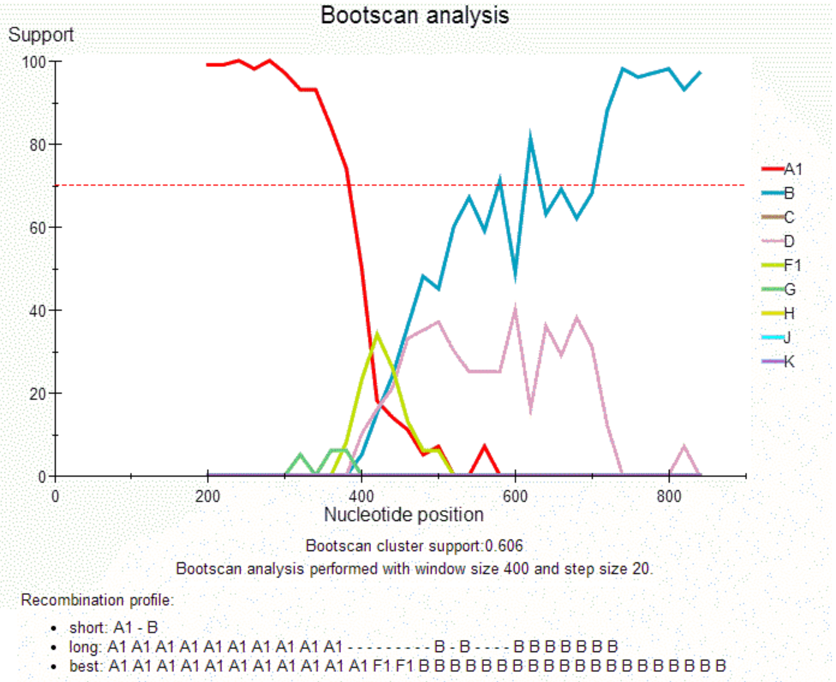

Supplement: S4 Fig — Four examples of recombinant viruses found among HIV infected patients. Recombinants between established subtypes were identified using the REGA Subtyping Tool [15] and SimPlot [16]. (PDF) [file pone.0135061.s004.pdf]
