# Supplementary material for: A Population-Structured HIV Epidemic in Israel: Roles of Risk and Ethnicity
Source: PLoS One. 2015 Aug 24;10(8):e0135061. doi: 10.1371/journal.pone.0135061 (PMC4547742; doi:10.1371/journal.pone.0135061)
Supplement: S1 Table — Characteristics of potential transmission networks embedded in larger clusters. (DOCX) [file pone.0135061.s005.docx]

**S1 Table : Transmission networks**

| **Cluster No.** | **Cluster size** | **Transmission network within the group** | **Members (n)** | **Node age (MRCA)** | **Composition (percent MSM)** | **Percent Female** | **Median Age** |
| --- | --- | --- | --- | --- | --- | --- | --- |
| **1** | **125** |  |  |  |  |  |  |
|  |  | **A** | **10** | **5.18** | **100%** | **0%** | **22.3** |
|  |  | **B** | **3** | **3.11** | **100%** | **0%** | **22.7** |
|  |  | **C** | **3** | **3.9** | **100%** | **0%** | **25.9** |
|  |  | **D** | **4** | **3.52** | **100%** | **0%** | **32.6** |
|  |  | **E** | **3** | **2.82** | **100%** | **0%** | **29.1** |
| **22** | **71** |  |  |  |  |  |  |
|  |  | **A** | **7** | **6.98** | **71%** | **0%** | **24.8** |
|  |  | **B** | **6** | **11.47** | **100%** | **0%** | **24.7** |
|  |  | **C** | **6** | **6.14** | **100%** | **0%** | **35.1** |
|  |  | **D** | **10** | **9.62** | **90%** | **0%** | **35.0** |
|  |  | **E** | **9** | **7.43** | **89%** | **0%** | **34.1** |
|  |  | **F** | **6** | **6** | **67%** | **17%** | **40.9** |
| **36** | **54** |  |  |  |  |  |  |
|  |  | **A** | **12** | **6.08** | **100%** | **0%** | **26.9** |
|  |  | **B** | **4** | **3.42** | **75%** | **0%** | **25.8** |
|  |  | **C** | **8** | **1.9** | **89%** | **0%** | **40.7** |
| **2** | **49** |  |  |  |  |  |  |
|  |  | **A** | **10** | **5.03** | **100%** | **0%** | **34.6** |
|  |  | **B** | **7** | **8.57** | **100%** | **0%** | **35.6** |
|  |  | **C** | **3** | **4.77** | **100%** | **0%** | **45.1** |
| **52** | **48** |  |  |  |  |  |  |
|  |  | **A** | **6** | **3.09** | **100%** | **0%** | **38.1** |
|  |  | **B** | **3** | **3.3** | **100%** | **0%** | **37.0** |
|  |  | **C** | **3** | **4.59** | **100%** | **0%** | **34.4** |
|  |  | **D** | **3** | **5.62** | **100%** | **0%** | **31.1** |
| **58** | **29** |  |  |  |  |  |  |
|  |  | **A** | **4** | **3.1** | **100%** | **0%** | **40.4** |
| **38** | **25** |  |  |  |  |  |  |
|  |  | **A** | **14** | **11.37** | **100%** | **0%** | **35.0** |
|  |  | **B** | **6** | **5.88** | **50%** | **33%** | **34.2** |
|  |  | **C** | **5** | **9.98** | **80%** | **0%** | **34.6** |
| **48** | **25** |  |  |  |  |  |  |
|  |  | **A** | **20** | **7.32** | **95%** | **0%** | **35.2** |
| **33** | **19** |  |  |  |  |  |  |
|  |  | **A** | **6** | **8.95** | **100%** | **0%** | **33,8** |
|  |  | **B** | **4** | **11.95** | **100%** | **0%** | **49.5** |
|  |  | **C** | **9** | **9.95** | **100%** | **0%** | **37.6** |
| **25** | **14** |  |  |  |  |  |  |
|  |  | **A** | **3** | **1.62** | **100%** | **0%** | **35.5** |
| **23** | **12** |  |  |  |  |  |  |
|  |  | **A** | **11** | **7.54** | **64%** | **0%** | **42.3** |
|  |  | **B** | **3** | **4.65** | **100%** | **0%** | **31.0** |
| **10** | **9** |  |  |  |  |  |  |
|  |  | **A** | **7** | **5.2** | **71%** | **0%** | **27.2** |
| **39** | **8** |  |  |  |  |  |  |
|  |  | **A** | **3** | **2.98** | **100%** | **0%** | **33.4** |
| **49** | **8** |  |  |  |  |  |  |
|  |  | **A** | **6** | **3.09** | **100%** | **0%** | **32.6** |

Characteristics of potential transmission networks within larger clusters. Potential networks are singled out by their shorter branch lengths relative to other members of the cluster, indicating that their viruses are more closely related. Some transmission nets had members with ages that were significantly younger (median age of youngest net (Cluster 1, group A) 22.3 y; p=0.002) or older (median age of the oldest net (Cluster 33, group B) 49.5 y; p=0.02) than the overall subtype-B infected population (34.3 y), suggesting networks with distinct characteristics.
