# Supplementary material for: A Population-Structured HIV Epidemic in Israel: Roles of Risk and Ethnicity
Source: PLoS One. 2015 Aug 24;10(8):e0135061. doi: 10.1371/journal.pone.0135061 (PMC4547742; doi:10.1371/journal.pone.0135061)
Supplement: S2 Table — Accessory mutations and polymorphisms listed by subtype. (DOCX) [file pone.0135061.s006.docx]

**S**2 **Table : Resistance related mutations**

|  | | **A** | | **B** | | **C** | | **Total** | | **P** | | |
| --- | --- | --- | --- | --- | --- | --- | --- | --- | --- | --- | --- | --- |
|  |  | **n=232** | | **n=770** | | **n=425** | | **n=1427** | | **A *vs*. B** | **B *vs*. C** | **A *vs*. C** |
|  |  | **No** | **%** | **No** | **%** | **No** | **%** | **No** | **%** |  |  |  |
| **PI** | **L10F** | **3** | **1.30%** | **4** | **0.50%** | **6** | **1.40%** | **13** | **0.90%** |  |  |  |
|  | **L10IV** | **38** | **16.40%** | **129** | **16.80%** | **21** | **4.90%** | **188** | **13.20%** | **1** | **<.0001** | **<.0001** |
|  | **V11IV** | **1** | **0.40%** | **2** | **0.30%** | **4** | **0.90%** | **7** | **0.50%** |  |  |  |
|  | **K20I** | **4** | **1.70%** | **5** | **0.60%** | **3** | **0.70%** | **12** | **0.80%** |  |  |  |
|  | **L24F** |  |  |  |  | **1** | **0.20%** | **1** | **0.10%** |  |  |  |
|  | **D30EY** |  |  |  |  | **2** | **0.50%** | **2** | **0.10%** |  |  |  |
|  | **V32A** |  |  |  |  | **1** | **0.20%** | **1** | **0.10%** |  |  |  |
|  | **L33F** |  |  | **4** | **0.50%** | **2** | **0.50%** | **6** | **0.40%** |  |  |  |
|  | **L33I** |  |  | **3** | **0.40%** |  |  | **3** | **0.20%** |  |  |  |
|  | **K43T** | **1** | **0.40%** | **2** | **0.30%** | **1** | **0.20%** | **4** | **0.30%** |  |  |  |
|  | **M46V** |  |  |  |  | **1** | **0.20%** | **1** | **0.10%** |  |  |  |
|  | **I47K** |  |  |  |  | **1** | **0.20%** | **1** | **0.10%** |  |  |  |
|  | **I47d** |  |  | **1** | **0.10%** |  |  | **1** | **0.10%** |  |  |  |
|  | **G48GW** | **1** | **0.40%** |  |  |  |  | **1** | **0.10%** |  |  |  |
|  | **Q58EQ** |  |  | **9** | **1.20%** | **1** | **0.20%** | **10** | **0.70%** |  |  |  |
|  | **A71ITV** |  |  | **186** | **24.20%** | **6** | **1.40%** | **192** | **13.50%** | **<.0001** | **<.0001** | **0.07** |
|  | **G73GV** |  |  | **1** | **0.10%** |  |  | **1** | **0.10%** |  |  |  |
|  | **T74S** | **16** | **6.90%** |  |  | **23** | **5.40%** | **39** | **2.70%** | **<.0001** | **<.0001** | **0.4** |
|  | **L76PQ** |  |  |  |  | **1** | **0.20%** | **1** | **0.10%** |  |  |  |
|  | **L90F** |  |  |  |  | **1** | **0.20%** | **1** | **0.10%** |  |  |  |
| **NRTI** | **A62V** | **32** | **13.80%** |  |  | **2** | **0.50%** | **34** | **2.40%** | **<.0001** | **<.0001** | **<.0001** |
|  | **K65N** |  |  | **2** | **0.30%** |  |  | **2** | **0.10%** |  |  |  |
|  | **T69N** | **2** | **0.90%** | **6** | **0.80%** |  |  | **8** | **0.60%** |  |  |  |
|  | **K70NTQ** |  |  | **2** | **0.30%** | **3** | **0.70%** | **5** | **0.40%** |  |  |  |
|  | **L74S** |  |  |  |  | **1** | **0.20%** | **1** | **0.10%** |  |  |  |
|  | **V75I** |  |  | **1** | **0.10%** |  |  | **1** | **0.10%** |  |  |  |
| **NNRTI** | **V90I** | **5** | **2.20%** | **8** | **1.00%** | **6** | **1.40%** | **19** | **1.30%** | **0.2** | **0.6** | **0.5** |
|  | **A98G** |  |  | **2** | **0.30%** | **11** | **2.60%** | **13** | **0.90%** |  |  |  |
|  | **L100FL** |  |  |  |  | **2** | **0.50%** | **2** | **0.10%** |  |  |  |
|  | **K103KT** | **1** | **0.40%** |  |  | **2** | **0.50%** | **3** | **0.20%** |  |  |  |
|  | **V106I** | **3** | **1.30%** | **23** | **3.00%** | **1** | **0.20%** | **27** | **1.90%** |  |  |  |
|  | **V108I** | **4** | **1.70%** | **1** | **0.10%** | **2** | **0.50%** | **7** | **0.50%** |  |  |  |
|  | **E138A** | **14** | **6.00%** | **18** | **2.30%** | **14** | **3.30%** | **46** | **3.20%** | **<0.005** | **0.3** | **0.1** |
|  | **V179D, E, T** | **4** | **1.70%** | **7** | **0.90%** | **4** | **0.90%** | **15** | **1.10%** |  |  |  |
|  | **Y181S, H** |  |  |  |  | **4** | **0.90%** | **4** | **0.30%** |  |  |  |
|  | **Y188D** |  |  |  |  | **1** | **0.20%** | **1** | **0.10%** |  |  |  |
|  | **H221Y** |  |  | **2** | **0.30%** | **1** | **0.20%** | **3** | **0.20%** |  |  |  |
|  | **F227FL** | **1** | **0.40%** |  |  |  |  | **1** | **0.10%** |  |  |  |
|  | **K238NT** | **2** | **0.90%** |  |  | **2** | **0.50%** | **4** | **0.30%** |  |  |  |

Accessory mutations and polymorphisms were listed by subtype. The frequency of the RT mutation A62V was 14% in A and 0% and 0.5% in B and C, respectively (p<0.0001); A98G appeared only in 3% of subtype-C sequences (*p*<0.02 and 0.006, for C vs. A and B, respectively); and E138A/G/Q tends to appear more in subtype A (6%) than in B or C (3% in each; *p*=0.04 and 0.06, respectively), and in 20% of the cases together with K103N (significantly more frequently than expected; *p*<0.0002). Pr L10V (particularly in subtype B with or without L90M) and E138A/G/Q (particularly in subtype A with or without K103N/S) were associated with clustering. A62V was the only mutation that appeared significantly more frequently among non-clustered sequences (*p*=0.01).
