# Supplementary material for: A Population-Structured HIV Epidemic in Israel: Roles of Risk and Ethnicity
Source: PLoS One. 2015 Aug 24;10(8):e0135061. doi: 10.1371/journal.pone.0135061 (PMC4547742; doi:10.1371/journal.pone.0135061)
Supplement: S1 Text — Accession numbers of sequences of Israeli patients and reference sequences from the Los Alamos database used in this study. (DOCX) [file pone.0135061.s007.docx]

**S1 Text: Accession numbers**

Accession numbers of sequences from Israeli patients submitted earlier:

[AY529528](http://www.hiv.lanl.gov/components/sequence/HIV/asearch/query_one.comp?se_id=AY529528)-[AY529627](http://www.hiv.lanl.gov/components/sequence/HIV/asearch/query_one.comp?se_id=AY529627) [[12](#_ENREF_12)]; [AY900811](http://www.hiv.lanl.gov/components/sequence/HIV/asearch/query_one.comp?se_id=AY900811)-AY900820, [AY901181](http://www.hiv.lanl.gov/components/sequence/HIV/asearch/query_one.comp?se_id=AY901181)-[AY901184](http://www.hiv.lanl.gov/components/sequence/HIV/asearch/query_one.comp?se_id=AY901184), [AY901353](http://www.hiv.lanl.gov/components/sequence/HIV/asearch/query_one.comp?se_id=AY901353)-[AY901365,](http://www.hiv.lanl.gov/components/sequence/HIV/asearch/query_one.comp?se_id=AY901353) AY901737-AY901786 [[30](#_ENREF_12)]; GQ398869, GQ398891, GQ398954, GQ399037, [GQ399057](http://www.hiv.lanl.gov/components/sequence/HIV/asearch/query_one.comp?se_id=GQ399057), [GQ399074](http://www.hiv.lanl.gov/components/sequence/HIV/asearch/query_one.comp?se_id=GQ399074), [GQ399060](http://www.hiv.lanl.gov/components/sequence/HIV/asearch/query_one.comp?se_id=GQ399074), GQ399123, GQ399133, [GQ399205,](http://www.hiv.lanl.gov/components/sequence/HIV/asearch/query_one.comp?se_id=GQ399074) [GQ399213,](http://www.hiv.lanl.gov/components/sequence/HIV/asearch/query_one.comp?se_id=GQ399074) [GQ399238,](http://www.hiv.lanl.gov/components/sequence/HIV/asearch/query_one.comp?se_id=GQ399074) [GQ399282,](http://www.hiv.lanl.gov/components/sequence/HIV/asearch/query_one.comp?se_id=GQ399074) [GQ399286,](http://www.hiv.lanl.gov/components/sequence/HIV/asearch/query_one.comp?se_id=GQ399074) [GQ399297,](http://www.hiv.lanl.gov/components/sequence/HIV/asearch/query_one.comp?se_id=GQ399074) [GQ399299,](http://www.hiv.lanl.gov/components/sequence/HIV/asearch/query_one.comp?se_id=GQ399074) [GQ399314,](http://www.hiv.lanl.gov/components/sequence/HIV/asearch/query_one.comp?se_id=GQ399074) [GQ399387,](http://www.hiv.lanl.gov/components/sequence/HIV/asearch/query_one.comp?se_id=GQ399074) [GQ399458,](http://www.hiv.lanl.gov/components/sequence/HIV/asearch/query_one.comp?se_id=GQ399074) [GQ399488,](http://www.hiv.lanl.gov/components/sequence/HIV/asearch/query_one.comp?se_id=GQ399074) [GQ399500,](http://www.hiv.lanl.gov/components/sequence/HIV/asearch/query_one.comp?se_id=GQ399074) [GQ399547,](http://www.hiv.lanl.gov/components/sequence/HIV/asearch/query_one.comp?se_id=GQ399074) [GQ399561,](http://www.hiv.lanl.gov/components/sequence/HIV/asearch/query_one.comp?se_id=GQ399074) [GQ399660,](http://www.hiv.lanl.gov/components/sequence/HIV/asearch/query_one.comp?se_id=GQ399074) [GQ399676,](http://www.hiv.lanl.gov/components/sequence/HIV/asearch/query_one.comp?se_id=GQ399074) [GQ399782,](http://www.hiv.lanl.gov/components/sequence/HIV/asearch/query_one.comp?se_id=GQ399074) [GQ399881,](http://www.hiv.lanl.gov/components/sequence/HIV/asearch/query_one.comp?se_id=GQ399074) [GQ399910,](http://www.hiv.lanl.gov/components/sequence/HIV/asearch/query_one.comp?se_id=GQ399074) [GQ399948,](http://www.hiv.lanl.gov/components/sequence/HIV/asearch/query_one.comp?se_id=GQ399074) [GQ400010,](http://www.hiv.lanl.gov/components/sequence/HIV/asearch/query_one.comp?se_id=GQ399074) [GQ400091,](http://www.hiv.lanl.gov/components/sequence/HIV/asearch/query_one.comp?se_id=GQ399074) [GQ400129,](http://www.hiv.lanl.gov/components/sequence/HIV/asearch/query_one.comp?se_id=GQ399074) [GQ400232,](http://www.hiv.lanl.gov/components/sequence/HIV/asearch/query_one.comp?se_id=GQ399074) [GQ400328,](http://www.hiv.lanl.gov/components/sequence/HIV/asearch/query_one.comp?se_id=GQ399074) [GQ400373,](http://www.hiv.lanl.gov/components/sequence/HIV/asearch/query_one.comp?se_id=GQ399074) [GQ400416,](http://www.hiv.lanl.gov/components/sequence/HIV/asearch/query_one.comp?se_id=GQ399074) [GQ400425,](http://www.hiv.lanl.gov/components/sequence/HIV/asearch/query_one.comp?se_id=GQ399074) [GQ400435,](http://www.hiv.lanl.gov/components/sequence/HIV/asearch/query_one.comp?se_id=GQ399074) [GQ400445,](http://www.hiv.lanl.gov/components/sequence/HIV/asearch/query_one.comp?se_id=GQ399074) [GQ400451,](http://www.hiv.lanl.gov/components/sequence/HIV/asearch/query_one.comp?se_id=GQ399074) [GQ400463,](http://www.hiv.lanl.gov/components/sequence/HIV/asearch/query_one.comp?se_id=GQ399074) GQ400467 [[11](#_ENREF_11)]; [JX299537](http://www.hiv.lanl.gov/components/sequence/HIV/asearch/query_one.comp?se_id=JX299537), [JX299544](http://www.hiv.lanl.gov/components/sequence/HIV/asearch/query_one.comp?se_id=JX299537), [JX299584](http://www.hiv.lanl.gov/components/sequence/HIV/asearch/query_one.comp?se_id=JX299537), [JX299598](http://www.hiv.lanl.gov/components/sequence/HIV/asearch/query_one.comp?se_id=JX299537), [JX299613](http://www.hiv.lanl.gov/components/sequence/HIV/asearch/query_one.comp?se_id=JX299537), [JX299625](http://www.hiv.lanl.gov/components/sequence/HIV/asearch/query_one.comp?se_id=JX299537), [JX299662](http://www.hiv.lanl.gov/components/sequence/HIV/asearch/query_one.comp?se_id=JX299537), [JX299679](http://www.hiv.lanl.gov/components/sequence/HIV/asearch/query_one.comp?se_id=JX299537), [JX299703](http://www.hiv.lanl.gov/components/sequence/HIV/asearch/query_one.comp?se_id=JX299537), [JX299726](http://www.hiv.lanl.gov/components/sequence/HIV/asearch/query_one.comp?se_id=JX299537), [JX299752](http://www.hiv.lanl.gov/components/sequence/HIV/asearch/query_one.comp?se_id=JX299537), [JX299756](http://www.hiv.lanl.gov/components/sequence/HIV/asearch/query_one.comp?se_id=JX299537), [JX299771](http://www.hiv.lanl.gov/components/sequence/HIV/asearch/query_one.comp?se_id=JX299537), [JX299775](http://www.hiv.lanl.gov/components/sequence/HIV/asearch/query_one.comp?se_id=JX299537), [JX299780](http://www.hiv.lanl.gov/components/sequence/HIV/asearch/query_one.comp?se_id=JX299537), [JX299781](http://www.hiv.lanl.gov/components/sequence/HIV/asearch/query_one.comp?se_id=JX299537), [JX299784](http://www.hiv.lanl.gov/components/sequence/HIV/asearch/query_one.comp?se_id=JX299537), [JX299794](http://www.hiv.lanl.gov/components/sequence/HIV/asearch/query_one.comp?se_id=JX299537), [JX299814](http://www.hiv.lanl.gov/components/sequence/HIV/asearch/query_one.comp?se_id=JX299537), [JX299840](http://www.hiv.lanl.gov/components/sequence/HIV/asearch/query_one.comp?se_id=JX299537), [JX299849](http://www.hiv.lanl.gov/components/sequence/HIV/asearch/query_one.comp?se_id=JX299537), [JX299851](http://www.hiv.lanl.gov/components/sequence/HIV/asearch/query_one.comp?se_id=JX299537), [JX299874](http://www.hiv.lanl.gov/components/sequence/HIV/asearch/query_one.comp?se_id=JX299537), [JX299922](http://www.hiv.lanl.gov/components/sequence/HIV/asearch/query_one.comp?se_id=JX299537), [JX299928](http://www.hiv.lanl.gov/components/sequence/HIV/asearch/query_one.comp?se_id=JX299537), [JX299945](http://www.hiv.lanl.gov/components/sequence/HIV/asearch/query_one.comp?se_id=JX299537), [JX299968](http://www.hiv.lanl.gov/components/sequence/HIV/asearch/query_one.comp?se_id=JX299537), [JX299971](http://www.hiv.lanl.gov/components/sequence/HIV/asearch/query_one.comp?se_id=JX299537), [JX299978](http://www.hiv.lanl.gov/components/sequence/HIV/asearch/query_one.comp?se_id=JX299537), [JX300040](http://www.hiv.lanl.gov/components/sequence/HIV/asearch/query_one.comp?se_id=JX299537), [JX300054](http://www.hiv.lanl.gov/components/sequence/HIV/asearch/query_one.comp?se_id=JX299537), [JX300075](http://www.hiv.lanl.gov/components/sequence/HIV/asearch/query_one.comp?se_id=JX299537), [JX300094](http://www.hiv.lanl.gov/components/sequence/HIV/asearch/query_one.comp?se_id=JX299537), [JX300](http://www.hiv.lanl.gov/components/sequence/HIV/asearch/query_one.comp?se_id=JX299537)105, [JX300](http://www.hiv.lanl.gov/components/sequence/HIV/asearch/query_one.comp?se_id=JX299537)109, [JX300](http://www.hiv.lanl.gov/components/sequence/HIV/asearch/query_one.comp?se_id=JX299537)126, [JX300](http://www.hiv.lanl.gov/components/sequence/HIV/asearch/query_one.comp?se_id=JX299537)159, [JX300](http://www.hiv.lanl.gov/components/sequence/HIV/asearch/query_one.comp?se_id=JX299537)170, [JX300](http://www.hiv.lanl.gov/components/sequence/HIV/asearch/query_one.comp?se_id=JX299537)194, [JX300](http://www.hiv.lanl.gov/components/sequence/HIV/asearch/query_one.comp?se_id=JX299537)199, [JX300](http://www.hiv.lanl.gov/components/sequence/HIV/asearch/query_one.comp?se_id=JX299537)215, [JX300](http://www.hiv.lanl.gov/components/sequence/HIV/asearch/query_one.comp?se_id=JX299537)216, [JX300](http://www.hiv.lanl.gov/components/sequence/HIV/asearch/query_one.comp?se_id=JX299537)273, [JX300](http://www.hiv.lanl.gov/components/sequence/HIV/asearch/query_one.comp?se_id=JX299537)278, [JX300](http://www.hiv.lanl.gov/components/sequence/HIV/asearch/query_one.comp?se_id=JX299537)295, [JX300](http://www.hiv.lanl.gov/components/sequence/HIV/asearch/query_one.comp?se_id=JX299537)298, [JX300](http://www.hiv.lanl.gov/components/sequence/HIV/asearch/query_one.comp?se_id=JX299537)332, [JX300](http://www.hiv.lanl.gov/components/sequence/HIV/asearch/query_one.comp?se_id=JX299537)374, [JX300](http://www.hiv.lanl.gov/components/sequence/HIV/asearch/query_one.comp?se_id=JX299537)381, [JX300](http://www.hiv.lanl.gov/components/sequence/HIV/asearch/query_one.comp?se_id=JX299537)394, [JX300](http://www.hiv.lanl.gov/components/sequence/HIV/asearch/query_one.comp?se_id=JX299537)536, [JX300](http://www.hiv.lanl.gov/components/sequence/HIV/asearch/query_one.comp?se_id=JX299537)544, [JX300](http://www.hiv.lanl.gov/components/sequence/HIV/asearch/query_one.comp?se_id=JX299537)551-[JX300](http://www.hiv.lanl.gov/components/sequence/HIV/asearch/query_one.comp?se_id=JX299537)553, [JX300](http://www.hiv.lanl.gov/components/sequence/HIV/asearch/query_one.comp?se_id=JX299537)571, [JX300](http://www.hiv.lanl.gov/components/sequence/HIV/asearch/query_one.comp?se_id=JX299537)577, [JX300](http://www.hiv.lanl.gov/components/sequence/HIV/asearch/query_one.comp?se_id=JX299537)582, [JX300](http://www.hiv.lanl.gov/components/sequence/HIV/asearch/query_one.comp?se_id=JX299537)588, [JX300](http://www.hiv.lanl.gov/components/sequence/HIV/asearch/query_one.comp?se_id=JX299537)617, [JX300](http://www.hiv.lanl.gov/components/sequence/HIV/asearch/query_one.comp?se_id=JX299537)673, [JX300](http://www.hiv.lanl.gov/components/sequence/HIV/asearch/query_one.comp?se_id=JX299537)679, [JX300](http://www.hiv.lanl.gov/components/sequence/HIV/asearch/query_one.comp?se_id=JX299537)682, [JX300](http://www.hiv.lanl.gov/components/sequence/HIV/asearch/query_one.comp?se_id=JX299537)687, [JX300](http://www.hiv.lanl.gov/components/sequence/HIV/asearch/query_one.comp?se_id=JX299537)720, [JX300](http://www.hiv.lanl.gov/components/sequence/HIV/asearch/query_one.comp?se_id=JX299537)721, [JX300](http://www.hiv.lanl.gov/components/sequence/HIV/asearch/query_one.comp?se_id=JX299537)738, [JX300](http://www.hiv.lanl.gov/components/sequence/HIV/asearch/query_one.comp?se_id=JX299537)751, [JX300](http://www.hiv.lanl.gov/components/sequence/HIV/asearch/query_one.comp?se_id=JX299537)754, [JX300](http://www.hiv.lanl.gov/components/sequence/HIV/asearch/query_one.comp?se_id=JX299537)771, [JX300](http://www.hiv.lanl.gov/components/sequence/HIV/asearch/query_one.comp?se_id=JX299537)781, [JX300](http://www.hiv.lanl.gov/components/sequence/HIV/asearch/query_one.comp?se_id=JX299537)790, [JX300](http://www.hiv.lanl.gov/components/sequence/HIV/asearch/query_one.comp?se_id=JX299537)840, [JX300](http://www.hiv.lanl.gov/components/sequence/HIV/asearch/query_one.comp?se_id=JX299537)841, [JX300](http://www.hiv.lanl.gov/components/sequence/HIV/asearch/query_one.comp?se_id=JX299537)968, [JX300](http://www.hiv.lanl.gov/components/sequence/HIV/asearch/query_one.comp?se_id=JX299537)975, [JX300](http://www.hiv.lanl.gov/components/sequence/HIV/asearch/query_one.comp?se_id=JX299537)985, [JX300](http://www.hiv.lanl.gov/components/sequence/HIV/asearch/query_one.comp?se_id=JX299537)986, JX301042 [[29](#_ENREF_29)]; KC184162-KC184396 [[3](#_ENREF_3)]; KC213492- KC213730 [[2](#_ENREF_2)]; KC218996-KC219037 [[30](#_ENREF_30)]; KF134929-KF135178 [[31](#_ENREF_31)].

Accession numbers of Israeli sequences submitted with this report: KM984894 - KM985369

Accession numbers of reference sequences from the Los Alamos database:

AB023804, AB032740, AB097871, AB098332, AB253421, AB254141, AB254149, AB485645, AB485645, AF069671, AF110959, AF193275, AF286223, AF286224, AF286227, AF286228, AF286233, AF286234, AF286237, AF400676, AF411966, AF413968, AF413987, AF443081, AF539405, AJ286980, AJ634690, AM000053, AY049708, AY118165, AY173959, AY180905, AY322189, AY500393, AY694336, AY713417, AY829205, AY829209, B097870, B221005, B428551, B485641, D10112, DQ207944, DQ275659, DQ39640, DQ67687, DQ823356, DQ823360, DQ823365, DQ823367, DQ878217, DQ878434, DQ878555, EF469243, EF514713, EF545108, EF589042, EU786673, EU786681, EU861977, F086817, F224507, F514698, F637046, F932474, FJ388901, FJ670521, FJ864679, FN557309, GQ999972, GU945074, HQ115067, HQ115068, HQ115070, HQ115071, HQ456677, IA04321, J388890, JF683803, JN687650, JQ292891, JQ292892, JQ292893, JQ292896, JQ403019, JQ403028, JQ779098, JQ779148, JQ779207, JQ779285, JX140651, JX140662, JX140663, JX140664, JX140665, JX140667, JX140669, JX500694, JX500695, JX500696, K03455, KA34828, KC156179, KC898980, KC898995, KF526210, KF716467, KF716492, KF835522, M030560, N248354, Q672623, Q853452, Q854714, U46016, U52953, U839600, Y037270, Y173951, Y423381, Y561236, Y682547, Y713408) [http://www.hiv.lanl.gov/].
